# Analysis and comparative study of a deterministic mathematical model of SARS-COV-2 with fractal-fractional operators: a case study

**DOI:** 10.1038/s41598-024-56557-6

**Published:** 2024-03-18

**Authors:** Khadija Tul Kubra, Rooh Ali, Rubayyi Turki Alqahtani, Samra Gulshan, Zahoor Iqbal

**Affiliations:** 1 Department of Mathematics, Government College University, Faisalabad, Punjab, 38040 Pakistan; 2 Department of Mathematics and Statistics, College of Science, Imam Mohammad Ibn Saud Islamic University(IMSIU), Riyadh, Saudi Arabia; 3 School of Computer Science and Technology, Zhejiang Normal University, Jinhua, 321004 China

**Keywords:** Epidemic model, Fractal-fractional model, SARS-CoV-2, Data fitting, Parameters estimation, Comparison, Applied mathematics, Scientific data, Software, Differential equations, Dynamical systems

## Abstract

In this paper, we investigate a fractal-fractional-order mathematical model with the influence of hospitalized patients and the impact of vaccination with fractal-fractional operators. The respective derivatives are considered in the Caputo, Caputo Fabrizio, and Atangana–Baleanu senses of fractional order $\alpha$ and fractal dimension $\tau$. For the proposed problem, some results regarding basic reproduction number and stability are given. Using the next-generation matrix approach, we have investigated the global and local stability of several types of equilibrium points. We provide a detailed analysis of the existence and uniqueness of the solution. Moreover, we fit the model with the real data of Pakistan from June 01, 2020, till March 24, 2021. Then, we use the fractal-fractional derivative to find a numerical solution for the model. MATLAB software is used for numerical illustration. Graphical presentations corresponding to different parameteric values are given as well.

## Introduction

The global impact of the COVID-19 pandemic that began in late 2019 has been unprecedented, affecting nearly every region of the world. Because it spread so quickly, the World Health Organization (WHO) called it a pandemic. Millions of people got sick and died around the world^1^. Global governments responded by enforcing strict lockdown rules, social distance guidelines, and other safety measures to stop the virus from spreading^2,3^. Several countries, including the UK, the USA, China, and Germany, suffered substantial economic losses during the COVID-19 pandemic. These countries rushed to create COVID-19 vaccines, and even though some were able to effectively contain the outbreak through proactive measures^4^, the development and distribution of vaccines became a global priority.

Due to the fast spread of this highly contagious and infectious disease, mathematical models have become important for understanding how diseases work and planning how to treat them^5,6^. The disease has symptoms that range from fever and cough to severe respiratory distress and neurological problems^1,7^. In addition to helping scientists figure out how the disease will spread, these models have also shown how well different treatments, like vaccinations, work^8^. It is important to keep in mind, though, that there is no “right” mathematical model and that many different modeling methods have been used. The study’s goal is to find out if fractional ordinary differential equations (FDEs) can be used to model how COVID-19 and vaccinations work together. In this paper, we chose to use FDEs as their modeling method, but it’s important to be aware of other modeling methods and talk about the pros and cons of each.

Recently, Kubra et al.^9^ presented an Atangana–Baleanu derivative-based fractal-fractional order model for the monkeypox virus, offering insights into the dynamics of the virus through a mathematical framework. The study contributes to the understanding of virus behavior and aids in developing strategies for virus control and prevention. Addressing the dynamics of the COVID-19 pandemic, Shah et al.^10^ proposed a comprehensive fractal-fractional mathematical model for the transmission and control of the virus. In parallel, Li et al.^11,12^ introduced an innovative approach for modeling financial bubbles using the fractal-fractional derivative of the Caputo sense, contributing insights into the dynamics of three-agent financial bubbles. Furthermore, Fatima et al.^13,14^ focused on modeling the epidemic trend of the Middle Eastern respiratory syndrome coronavirus, incorporating optimal control strategies to minimize infections while keeping intervention costs low. Kubra and Ali, expanded this approach to the analysis of a novel COVID-19 outbreak in Pakistan, employing a fractal-fractional derivative in the Caputo sense with a power-law kernel^15^. These studies collectively showcase the versatility and applicability of fractal-fractional mathematical models in understanding and controlling complex phenomena across epidemiology and financial dynamics.

Using a deterministic mathematical model enhanced with fractal-fractional operators, this study delves into the complex dynamics of the SARS-CoV-2 outbreak in Pakistan. Deterministic modeling forms the foundation, providing a structured framework for understanding the transmission dynamics of the virus. This research significantly contributes to the broader conversation on pandemic modeling, complementing existing methods and fostering a comprehensive understanding of infectious disease dynamics. Additionally, it aims to compare its findings with related studies such as the investigation of financial bubble mathematical models under fractal-fractional Caputo derivative, modeling the epidemic trend of the Middle Eastern respiratory syndrome coronavirus with optimal control, predictive modeling and control strategies for the transmission of Middle East Respiratory Syndrome Coronavirus, dynamical properties of a meminductor chaotic system with fractal-fractional power law operator, and exploring the role of fractal-fractional operators in the mathematical modeling of corruption. The incorporation of fractal-fractional operators in these diverse contexts underscores the versatility and applicability of the proposed modeling approach.

In this context, this research aims to provide a fresh perspective by incorporating FDEs into the modeling framework. By doing so, it seeks to better understand the impact of vaccination on COVID-19 eradication in the population and accelerate recovery. To achieve this, the study evaluates the dynamics of COVID-19 cases in Pakistan over a specific time frame, ultimately comparing the model’s performance with reported cases to gain insights into disease propagation. This work contributes to the broader conversation surrounding modeling approaches for pandemic analysis, complementing existing methods and fostering a more comprehensive understanding of infectious disease dynamics.

The manuscript is presented in the following order: we discuss the fundamentals of advanced fractional calculus in “Preliminaries” and describe some of its core concepts. We provide a brief explanation of the mathematical modeling of the SARS-CoV-2 with the influence of hospitalized patients and the impact of vaccination in “Model formation”. We examined the model’s fundamental characteristics and conduct a “Qualitative analysis” of them. The proposed “Mathematical model in fractal-fractional sense”, is also given, and then for the proposed mathematical model the “Existence and uniqueness results for the solution” under Atangana–Baleanu fractal-fractional derivative is aslo discussed. For the “Fractal fractional derivative in Caputo sense, Fractal-fractional derivative in Caputo–Fabrizio sense, and Fractal-fractional derivative in Atangana–Baleanu sense”, we develop some new fractal fractional numerical schemes. Finally, a comparison for all of these operators is provided reffer to “Comparison”. We provide a graphical representation of the results for our proposed model, taking into account parameteric values from the literature and some of them being fitted. We further studied the simulations of “Results and discussion” for adjusting various parameters. In “Conclusion”, concluding remarks are given.

### Preliminaries

Now that we’ve recalled a few preliminary definitions that are crucial to understanding this paper, are briefly discussed in this subsection.

#### Definition 1.1

^16^ Suppose that $y \left( t \right)$ be differentiable in opened interval $\left( a, b \right)$ with order $\tau$ then the Fractal-Fractional derivative of $y \left( t \right)$ with fractional order $\eta$ in caputo sense having power law type kernel is defined as follows:

1 $$\begin{aligned} ^{FFP}D^{\eta , \tau }_{0, t} \left( y \left( t \right) \right)= & {} \frac{1}{\Gamma \left( m - \eta \right) } \int _{0}^{t} \frac{d y \left( s \right) }{ds^\tau } \left( t - s \right) ^{m - \eta - 1} ds, \end{aligned}$$

where $m - 1< \eta \le m, 0< m - 1 < \tau \le m$ and $\frac{d y \left( s \right) }{ds^\tau } = \lim _{t \rightarrow s} \frac{y \left( t \right) - y \left( s \right) }{t^{\tau } - s^{\tau }}$. The more generalized version is given as:

2 $$\begin{aligned} ^{FFP}D^{\eta , \tau }_{0, t} \left( y \left( t \right) \right)= & {} \frac{1}{\Gamma \left( m - \eta \right) } \int _{0}^{t} \frac{d^{\lambda } y \left( s \right) }{ds^\tau } \left( t - s \right) ^{m - \eta - 1} ds, \end{aligned}$$

where $$ m - 1< \eta \le m, 0< m - 1 < \lambda , \tau \le m $$ and $$\frac{d^{\lambda } y \left( s \right) }{ds^\tau } = \lim _{t \rightarrow s} \frac{y^{\lambda } \left( t \right) - y^{\lambda } \left( s \right) }{t^{\tau } - s^{\tau }}$$.

#### Definition 1.2

^16^ Suppose that $y \left( t \right)$ be continuous on an open interval $\left( a, b \right)$ then the fractal-fractional integral of $y \left( t \right)$ with order $\eta$ having power law type kernel is defined as follows:

3 $$\begin{aligned} ^{FFP}J^{\eta }_{0, t} \left( y \left( t \right) \right)= & {} \frac{\tau }{\Gamma \left( \eta \right) } \int _{0}^{t} \left( t - s \right) ^{\eta - 1} s^{\tau - 1} y \left( s \right) ds. \end{aligned}$$

#### Definition 1.3

^16^ Suppose that $y \left( t \right)$ be continuous and fractal differentiable on $\left( a, b \right)$ with order $\tau$ then the fractal-fractional derivative of $y \left( t \right)$ with order $\eta$ in the caputo sense having exponentially decaying type kernel is defined as follows:

4 $$\begin{aligned} ^{FFE}D^{\eta , \tau }_{0, t} \left( y \left( t \right) \right)= & {} \frac{M \left( \eta \right) }{1 - \eta } \int _{0}^{t} \frac{d y \left( s \right) }{ds^\tau } \exp \left( - \frac{\eta }{1 - \eta } \left( t - s \right) \right) ds, \end{aligned}$$

where $0 < \eta , \tau \le m$ and $M \left( 0 \right) = M \left( 1 \right) = 1$. The more generalized version is given as:

5 $$\begin{aligned} ^{FFE}D^{\eta , \tau , \lambda }_{0, t} \left( y \left( t \right) \right)= & {} \frac{M \left( \eta \right) }{1 - \eta } \int _{0}^{t} \frac{d y^{\lambda } \left( s \right) }{ds^\tau } \exp \left( - \frac{\eta }{1 - \eta } \left( t - s \right) \right) ds, \end{aligned}$$

where $$ 0 < \eta , \tau , \lambda \le m $$.

#### Definition 1.4

^16^ Suppose that $y \left( t \right)$ be continuous on an open interval $\left( a, b \right)$ then the fractal-fractional integral of $y \left( t \right)$ with order $\eta$ having exponentially decaying type kernel is defined as follows:

6 $$\begin{aligned} ^{FFE}J^{\eta , \tau }_{0, t} \left( y \left( t \right) \right)= & {} \frac{\eta \tau }{M \left( \eta \right) } \int _{0}^{t} s^{\eta - 1} y \left( s \right) ds + \frac{\tau \left( 1 - \eta \right) t^{\tau - 1} y \left( t \right) }{M \left( \eta \right) }. \end{aligned}$$

#### Definition 1.5

^16^ Suppose that $y \left( t \right)$ be continuous and fractal differentiable on $\left( a, b \right)$ with order $\tau$ then the fractal-fractional derivative of $y \left( t \right)$ with order $\eta$ in the Riemann–Liouville sense having generalized Mittag–Leffler type kernel is defined as follows:

7 $$\begin{aligned} ^{FFM}D^{\eta , \tau }_{0, t} \left( y \left( t \right) \right)= & {} \frac{AB \left( \eta \right) }{1 - \eta } \int _{0}^{t} \frac{d y \left( s \right) }{ds^\tau } E_\eta \left( - \frac{\eta }{1 - \eta } \left( t - s \right) ^{\eta } \right) ds, \end{aligned}$$

where $0 < \eta , \tau \le 1$ and $AB \left( \eta \right) = 1 - \eta + \frac{\eta }{\Gamma \left( \eta \right) }$. The more generalized version is given as:

8 $$\begin{aligned} ^{FFM}D^{\eta , \tau , \lambda }_{0, t} \left( y \left( t \right) \right)= & {} \frac{AB \left( \eta \right) }{1 - \eta } \int _{0}^{t} \frac{d y^{\lambda } \left( s \right) }{ds^\tau } E_\eta \left( - \frac{\eta }{1 - \eta } \left( t - s \right) ^{\eta } \right) ds, \end{aligned}$$

where $$ 0 < \eta , \tau , \lambda \le m $$.

#### Definition 1.6

^16^ Suppose that $y \left( t \right)$ be continuous on an open interval $\left( a, b \right)$ then the fractal-fractional integral of $y \left( t \right)$ with order $\eta$ having generalized Mittag-Leffler type kernel is defined as follows:

9 $$\begin{aligned} ^{FFM}J^{\eta , \tau }_{0, t} \left( y \left( t \right) \right)= & {} \frac{\eta \tau }{AB \left( \eta \right) } \int _{0}^{t} s^{\tau - 1} y \left( s \right) \left( t - s \right) ^{\eta - 1} ds + \frac{\tau \left( 1 - \eta \right) t^{\tau - 1} y \left( t \right) }{AB \left( \eta \right) }. \end{aligned}$$

## Model formation

**Figure 1:**
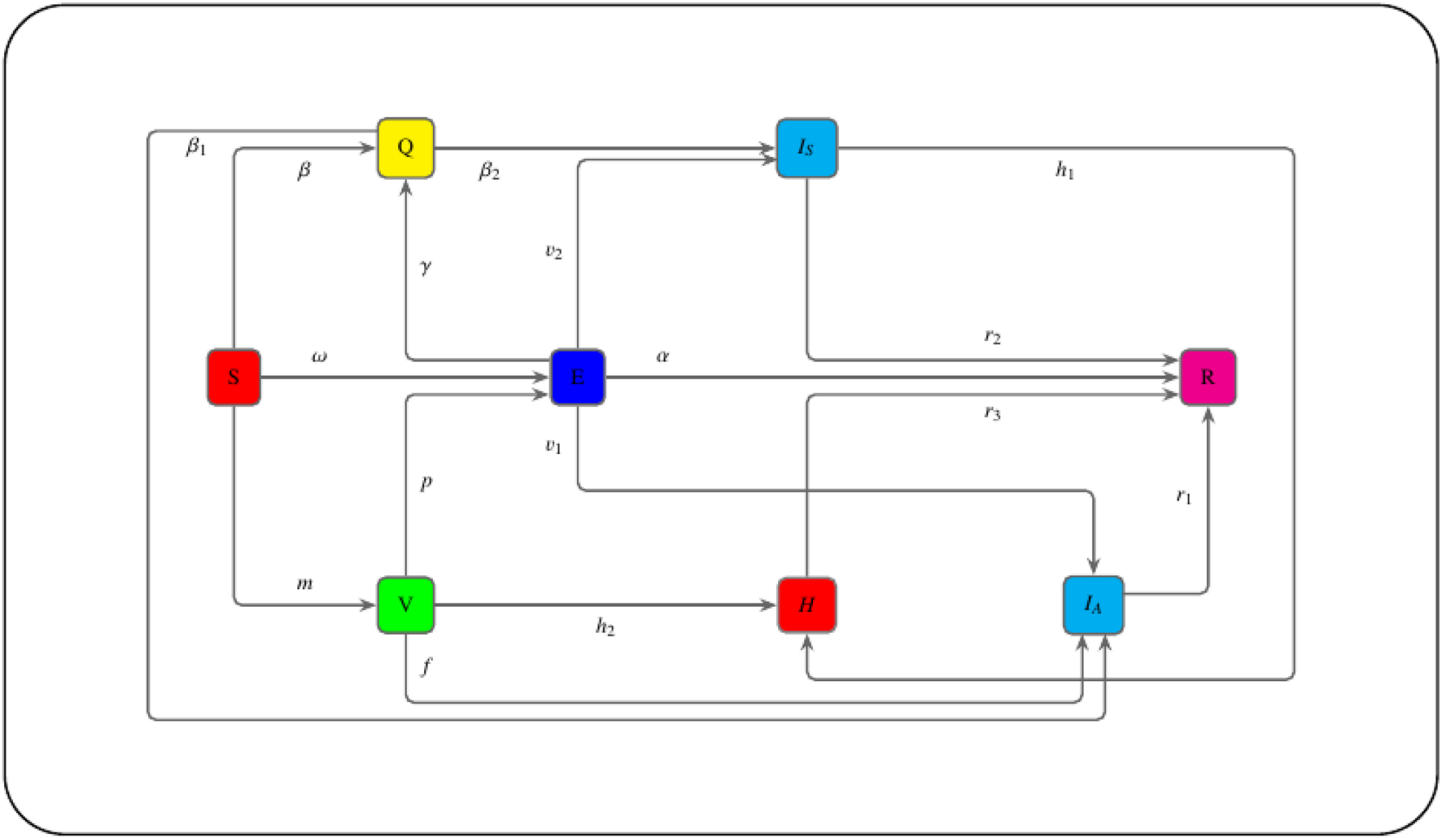
Dynamical phase diagram of COVID-19 model, transmitting impact of hospital treatment Included the third shot of vaccine.

In this research paper, we extended a compartmental model presented by Ahmed et al.^17^, with the impact of vaccination (see Fig. 1 for the model diagram). We account for the influence of hospitalized patients for a lower susceptibility to SARS-CoV-2 infection as well as third dose of vaccine called booster shot. The present mathematical model considered the epidemic wave of SARS-CoV-2. Our model explicitly accounted for the decay of vaccine effectiveness^18^. Due to the fact that symptomatic infected peoples are being hospitalized, this suggests that the entire population of individuals is $W \left( t \right)$, which would have been divided into eight classes: susceptible individuals, exposed individuals, quarantined individuals, symptomatically infected individuals, hospitalized individuals, asymptomatically infected individuals, and individuals that have take away from corona-virus. As a consequence of this, the total population $W \left( t \right) = S \left( t \right) + E \left( t \right) + I_A \left( t \right) + I_S \left( t \right) + H \left( t \right) + Q \left( t \right) + V \left( t \right) + R \left( t \right)$. Considering the discussion of the COVID-19 classes, Fig. 1 is where you may find the flow transmission through one compartment to another. This diagram illustrates the process of developing a system of nonlinear ordinary differential equations (for further information, see Table 1). If $\Pi ,$ and $\zeta$ are the natural natality and mortality rate respectively, then the proposed mathematical model is given as:

10 $$\begin{aligned} \displaystyle \frac{dS}{dt}= & {} \Pi - \omega ES - \left( \beta + m + \zeta \right) S, \nonumber \\ \displaystyle \frac{dE}{dt}= & {} p EV + \omega SE - \left( \gamma + \upsilon _{1} + \upsilon _{2} + \alpha + \zeta \right) E, \nonumber \\ \displaystyle \frac{dQ}{dt}= & {} \beta S + \gamma E - \left( \beta _{1} + \beta _{2} + \zeta \right) Q, \nonumber \\ \displaystyle \frac{dI_A}{dt}= & {} \upsilon _{1} E + f V + \beta _{1} Q - \left( r_{1} + \delta +\zeta \right) I_A, \nonumber \\ \displaystyle \frac{dI_S}{dt}= & {} \upsilon _{2} E +\beta _{2} Q - h_1 HI_S - \left( r_2 + \zeta \right) I_S, \nonumber \\ \displaystyle \frac{dH}{dt}= & {} h_1 HI_S - \left( h_2 + r_3 + \delta + \zeta \right) H, \nonumber \\ \displaystyle \frac{dV}{dt}= & {} m S + h_2 H - p EV - \left( f + \zeta \right) V, \nonumber \\ \displaystyle \frac{dR}{dt}= & {} \alpha E + r_1 I_A + r_2 I_S + r_3 H - \zeta R, \end{aligned}$$

with the initial conditions $S\left( 0\right) =S_0, E\left( 0\right) =E_0, Q\left( 0\right) =Q_0, I_A\left( 0\right) =I_{A_{0}}, I_S\left( 0\right) =I_{S_{0}}, H\left( 0\right) =H_0, V\left( 0\right) =V_0$ and $R\left( 0\right) =R_0$ respectively. Wherever $\left( S\left( t\right) , E\left( t\right) , Q\left( t\right) , I_A\left( t\right) , \right.$ $\left. I_S\left( t\right) , H\left( t\right) , Q\left( t\right) , V\left( t\right) , R\left( t\right) \right) \in {\mathscr {R}}^8_+$, the functions $S\left( t\right) , E\left( t\right) , Q\left( t\right) , I_A\left( t\right) , I_S\left( t\right) ,$ $H\left( t\right) , V\left( t\right) , R\left( t\right)$ and their derivatives are assumed to be continuous for $t \ge 0$.

### Model framework

Individuals who are susceptible receive their vaccination dosage at a rate of *m*, or they transfer to a class that is quarantined at a rate of $\beta$, or they may become infected as a result of sufficient interaction with individuals who are exposed at a rate of $\omega$. After vaccination, the peoples may be asymptomatically infected (this occurs when the vaccine is ineffective), and if this occurs, the individuals may move to an asymptomatically infected compartment with a rate of *f*. If this does not occur, the vaccine may be exposed to the virus at a rate of *p*. After being exposed to the virus, the individuals will be thrown in the quarantined class, or have a chance of becoming infected with or without symptoms, according to the rates denoted by $\gamma$, $\upsilon _2$, and $\upsilon _1$, respectively. Those individuals who are able to beat the condition after vaccination and become disease-free are promoted to the recovery class at the rate of $\alpha$. In addition, peoples who have been placed in a quarantine facility have a greater chance of being entirely infected by the virus as a result of the analysis, with a rate of $\beta _2$ for those who have symptoms and $\beta _1$ for those who do not show symptoms. Individuals who are symptomatically infected may have major health problems and be hospitalized with the rate of $h_1$ against the virus, after which they are injected with the first, second or third doses of vaccines with the rate of $h_2$, or they may be recovered with the rate of $r_3$, due to the best treatment in the hospital, depending on the severity of their symptoms. Recovery from the virus can occur in either asymptomatic infected peoples or symptomatic infected individuals, with the rates of recovery being $r_1$ and $r_2$ accordingly. Each of these subgroups of people has a chance of being smaller as a result of natural mortality at a rate of $\zeta$, but the class of people who are infected has a chance of becoming smaller as a consequence of mortality caused by the corona-virus at a rate of $\delta$. Therefore, the presumed natality rate, denoted by $\Pi$, and the mortality rate, denoted by $\zeta$, are respectively represented by these two values.

The success of the system is contingent not only on the mechanisms of virus infection but also on the presence or absence of virus exposure in individuals who have never been exposed to the virus or in those who are hospitalized with the best treatment according to the severity of the symptoms of the virus. We believe that it is important to clarify what we mean by booster shots. When it comes to vaccines, the actual question is: Is it necessary to take a third dosage if you’ve had your first two doses? We may want to recomend another dose for three different reasons. A third dose may be required for immunocompromised patients since the first two doses aren’t working as well as they should in otherwise healthy people. This is especially true if you didn’t respond or fall into a category of people who didn’t respond properly after receiving the first two doses. Secondly, if the immunity that you got and achieved as a result of vaccination begins to fade, it begins to diminish or decline over time. In reality, current research suggests that vaccinations are incredibly effective at keeping you safe from serious illness, hospitalization, or even death. People who have already received two doses of the vaccine do not appear to require another shot. A third dose may be necessary if the vaccines do not adequately protect against some of the new variants of COVID-19 that are causing us fear. The current vaccinations against the variations, which we’re monitoring very closely, are holding up quite well against the most severe forms of the disease.

**Table 1:** Discription of parameter for model (10).

Parameter	Interpretation	Value
$\beta$	The number of peoples who have been quarantined because they are susceptible is increasing.	0.0002^17^
$\omega$	Transition from sensitive to exposed peoples at a rapid rate	0.002^17^
$\gamma$	The rate at which exposed individuals are transferred to quarantine.	$2.0138 \times 10^{-4}$^17^
*m*	Proportion of susceptible people who have been vaccinated.	0.5^19^
$\beta _{2}$	High rates of moving people from the quarantined group to the symptomatic infected group.	$3.2084 \times 10^{-4}$^17^
*p*	Vaccinated people’s disease exposure rate.	0.08^17^
$\beta _{1}$	Quarantined people to asymptomatic infected people ratio.	0.0101
$\zeta$	Natural death rate.	0.009^19^
$\alpha$	Recovery rate of exposed individuals.	0.05^19^
$\delta$	Disease-related mortality.	0.25^17^
$r_{1}$	Recovery rate of asymptomatic infected individuals.	0.012^17^
$r_{2}$	Recovery rate of symptomatic infected individuals.	0.012
$r_{3}$	Recovery rate of symptomatic infected individuals who are in hospitalized.	0.072
$h_{1}$	Transfer rate of symptomatically infected individuals to hospital.	$3.2084 \times 10^{-4}$^17^
$h_{2}$	Proportion of hospitalized people who have been injected by booster dose.	0.021^17^

### Model fitted with real data

**Figure 2:**
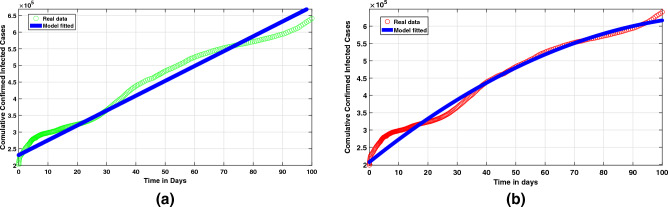
Data since June 01, 2020 to March 24, 2021 versus (**a**) model (10) fitting with integer-order derivative, and in (**b**) model (29) fitting with fractional-order derivative.

The main objective of this study is to identify optimal models that align with the reported cases in Pakistan^20^, followed by an examination of the impact of behavioral changes on the management of this emerging infectious disease. The best-fitting curve from models (10) and (29), together with its associated parameters, may be seen in Fig. 2. These models were chosen based on their ability to accurately capture the trend of reported cases in Pakistan. Additionally, the analysis will also explore how different interventions and control measures can affect the trajectory of the disease. Through the use of curve fitting techniques and real data from the COVID-19 outbreak in Pakistan^20^, the authors created an optimized epidemic model (10). Finally, we explain how these false assumptions about the structure of model (10) contribute to both the recovery of affected individuals and the maintenance of social welfare given the obstacles posed by these diseases. Furthermore, recommendations have been made regarding the possibility that the populace could eradicate viruses or efficiently control them by putting appropriate control measures in place.

## Qualitative analysis

In order to properly examine the infectious disease model, reproduction number is very important^21^. The positivity, invariant region and basic reproduction number for the proposed model are computed and presented in this section, deals with the study of stability of basic reproduction number in Theorem 3.1 at disease free equilibrium point, Theorem 3.2 deal with the existence of unique endemic equilibrium point, and Theorem 3.4 deal with the stability of its endemic equilibrium point.

### Positivity and biologically invariant region

The state parameters described by the model (10) must be shown to be non-negative for all $t \ge 0$ in order to establish their validity following the population description. The solution of the given problem, under the assumption of positive initial data, exhibits positivity for all time values greater than or equal to zero and is also bounded. Using the systems (10), it is simple to see $\frac{dW \left( t \right) }{dt} = \Pi - \zeta W \left( t \right) - \delta \left( I_A \left( t \right) + H \left( t \right) \right)$ and $\sup _{t \rightarrow + \infty } W \left( t \right) \le \frac{\Pi }{\zeta }$. This allows us to investigate the system (10) in the following tractable domain:

11 $$\begin{aligned} & {} \left\{ \left( S\left( t\right) ,\ E\left( t\right) ,\ Q\left( t\right) ,\ I_A\left( t\right) ,\ I_S\left( t\right) ,\ H\left( t\right) ,\ V\left( t\right) ,R\left( t\right) \right) \mathrm {\in }{\mathscr {R}}^8_+\ \Bigg \vert \ 0 \le \sup _{t \rightarrow + \infty } W\left( t\right) \mathrm {\le }\displaystyle \frac{\Pi }{\zeta }\right\} . \end{aligned}$$

The system (10) is now positively invariant with respect to Eq. (11). So, the proposed model (10) is properly posed from an epidemiological perspective, and the system has solutions with $\left( S\left( t\right) ,\ E\left( t\right) ,\ Q\left( t\right) ,\ I_A\left( t\right) ,\ I_S\left( t\right) ,\ H\left( t\right) ,\ V\left( t\right) ,R\left( t\right) \right) \mathrm {\in }{\mathscr {R}}^8_+$ remain in $\mathfrak {B}$^22^.

### Disease free equilibrium point

In epidemiology, the disease-free equilibrium point refers to a state in which a population is free from any infectious disease. At this point, there are no infected individuals within the population, and the disease does not spread further. This equilibrium is achieved when the rate of new infections is balanced by the rate of recovery or removal of infected individuals from the population. It represents a desirable goal for public health efforts and can be maintained through effective prevention measures such as vaccination campaigns and prompt identification and isolation of infected individuals. The system (10) denotes that the DFE point is determined by

12 $$\begin{aligned} \text {DFE}= & {} \left( S_0, E_0, Q_0, I_{A_0}, I_{S_0}, H_0, V_0, R_0 \right) = \left( \displaystyle \frac{\Pi }{\beta +m+\zeta },\ 0,\ 0,\ 0,\ 0,\ 0,\ \displaystyle \frac{m \Pi }{\left( \beta +m+\zeta \right) \left( f+\zeta \right) },\ 0\right) . \end{aligned}$$

### Basic reproduction number

The number corresponding to the infection rate predicted to occur per unit of time is referred to as the basic reproduction number ${\mathscr {R}}_0$. This infection spreads throughout the susceptible population as a result of a people who is already infected with it. The section creates a new system that is based on the system (10), and it includes the classes of exposed populations, infected populations (both asymptomatic and symptomatic populations), quarantined, and hospitalized populations . In these infected classes ${\mathscr {R}}_0$ for the mathematical model (10) is described. By utilizing the method of next generation matrices, the value ${\mathscr {R}}_0$ is discovered for the system (13), wherever other writers have used it in their work^21^. It is abundantly clear from the model (13) without sacrificing generality the article develops a system that has the following classifications of the exposed, infected, quarantined, and hospitalized population:

13 $$\begin{aligned} \displaystyle \frac{dE}{dt}= & {} p E V + \omega S E - \left( \gamma + \upsilon _{1} + \upsilon _{2} + \alpha + \zeta \right) E, \nonumber \\ \displaystyle \frac{dQ}{dt}= & {} \beta S + \gamma E - \left( \beta _{1} + \beta _{2} + \zeta \right) Q, \nonumber \\ \displaystyle \frac{dI_A}{dt}= & {} \upsilon _{1} E + f V + \beta _{1} Q - \left( r_{1} + \delta +\zeta \right) I_A, \nonumber \\ \displaystyle \frac{dI_S}{dt}= & {} \upsilon _{2} E +\beta _{2} Q - h_1 H I_S - \left( r_2 + \zeta \right) I_S, \nonumber \\ \displaystyle \frac{dH}{dt}= & {} h_1 H I_S - \left( h_2 + r_3 + \delta + \zeta \right) H, \end{aligned}$$

Referring to^23^, from the system (13), the study generates matrix $\textbf{F}$ and $\textbf{V}$, i.e.

$$ \begin{aligned} \textbf{F} = \left( \begin{array}{c} pEV+\omega SE \\ 0 \\ 0 \\ 0 \\ 0 \end{array} \right) \qquad  \&  \qquad \textbf{V} = \left( \begin{array}{c} -\left( \gamma + \upsilon _{1} + \upsilon _{2} + \alpha + \zeta \right) E \\ -\upsilon _{1} E - f V - \beta _{1} Q + \left( r_{1} + \delta +\zeta \right) I_A \\ -\upsilon _{2} E - \beta _{2} Q - h_1 H I_S + \left( r_2 + \zeta \right) I_S \\ - h_1 H I_S + \left( h_2 + r_3 + \delta + \zeta \right) H \\ - \beta S - \gamma E + \left( \beta _{1} + \beta _{2} + \zeta \right) Q \end{array} \right) , \end{aligned}$$

Here, $\texttt{F}$ and $\texttt{V}$ represents the jacobian matrix at DFE point for the matrix $\textbf{F}$ and $\textbf{V}$, and is written as:

$$\begin{aligned} \texttt{F}= & {} \left( \begin{array}{ccccc} \displaystyle \frac{\Pi \left( m p + \omega \left( f + \zeta \right) \right) }{\left( \beta + m + \zeta \right) \left( f+\zeta \right) } &{} 0 &{} 0 &{} 0 &{} 0 \\ 0 &{} 0 &{} 0 &{} 0 &{} 0 \\ 0 &{} 0 &{} 0 &{} 0 &{} 0 \\ 0 &{} 0 &{} 0 &{} 0 &{} 0 \\ 0 &{} 0 &{} 0 &{} 0 &{} 0 \end{array} \right) , \\ \texttt{V}= & {} \left( \begin{array}{ccccc} - \gamma - \upsilon _{1} - \upsilon _{2} - \alpha - \zeta &{} 0 &{} 0 &{} 0 &{} 0 \\ -{\upsilon }_1 &{} - r_1 - \delta - \zeta &{} 0 &{} 0 &{} - {\beta }_1 \\ -{\upsilon }_2 &{} 0 &{} - r_2 - \zeta &{} 0 &{} - {\beta }_2 \\ 0 &{} 0 &{} 0 &{} - h_2 - r_3 - \delta -\zeta &{} 0 \\ -\gamma &{} 0 &{} 0 &{} 0 &{} -{\beta }_1 - {\beta }_2 - \zeta \end{array} \right) , \end{aligned}$$

this implies that

$$\begin{aligned} \mathtt {FV^{-1}}= & {} \left( \begin{array}{ccccc} \displaystyle \frac{\Pi \left( m p + \omega \left( f + \zeta \right) \right) }{\left( \gamma +{\upsilon }_1+{\upsilon }_2+ \alpha +\zeta \right) \left( \beta +m+\zeta \right) \left( f+\zeta \right) } &{} 0 &{} 0 &{} 0 &{} 0 \\ 0 &{} 0 &{} 0 &{} 0 &{} 0 \\ 0 &{} 0 &{} 0 &{} 0 &{} 0 \\ 0 &{} 0 &{} 0 &{} 0 &{} 0 \\ 0 &{} 0 &{} 0 &{} 0 &{} 0 \end{array} \right) . \end{aligned}$$

So, $\varrho \left( \mathtt {FV^{-1}} \right) = \frac{\Pi \left( m p + \omega \left( f + \zeta \right) \right) }{ab\left( f+\zeta \right) } = \mathscr {R}_0$, where $a = \gamma +{\upsilon }_1+{\upsilon }_2+ \alpha +\zeta$ and $b = \beta + m + \zeta$, Therefore

14 $$\begin{aligned} \mathscr {R}_0= & {} \displaystyle \frac{\Pi \left( m p + \omega \left( f + \zeta \right) \right) }{\left( \gamma +{\upsilon }_1+{\upsilon }_2+ \alpha +\zeta \right) \left( \beta +m+\zeta \right) \left( f+\zeta \right) } \end{aligned}$$

### Local stability analysis of the disease free equilibrium

#### Theorem 3.1

*The disease free equilibrium DFE is locally asymptotically stable if* $\mathscr {R}_0 < 1$.

#### *Proof*

In direction to point out the stability conditions for the system (13) at the DFE point, let us consider the following Jacobian matrix $\mathscr {J}\left( \textrm{DFE}\right)$ with respect to the system (13),

$$\begin{aligned} \mathscr {J}(\textrm{DFE}) = \left( \begin{array}{cccccccc} -\beta - m - \zeta &{} -\frac{\omega \Pi }{\beta + m + \zeta } &{} 0 &{} 0 &{} 0 &{} 0 &{} 0 &{} 0 \\[10pt] 0 &{} \frac{\Pi (mp + \omega (f + \zeta )) - (\gamma + \upsilon _{1} + \upsilon _{2} + \alpha + \zeta )}{(\beta + m + \zeta )(f + \zeta )} &{} 0 &{} 0 &{} 0 &{} 0 &{} 0 &{} 0 \\[10pt] 0 &{} \upsilon _1 &{} -r_1 - \delta - \zeta &{} 0 &{} 0 &{} \beta _1 &{} f &{} 0 \\[10pt] 0 &{} \upsilon _2 &{} 0 &{} -r_2 - \zeta &{} 0 &{} \beta _2 &{} 0 &{} 0 \\[10pt] 0 &{} 0 &{} 0 &{} 0 &{} -h_2 - r_3 - \delta - \zeta &{} 0 &{} 0 &{} 0 \\[10pt] 0 &{} \gamma &{} 0 &{} 0 &{} 0 &{} -\beta _1 - \beta _2 - \zeta &{} 0 &{} 0 \\[10pt] m &{} \frac{-mp\Pi }{(\beta + m + \zeta )(f + \zeta )} &{} 0 &{} 0 &{} h_2 &{} 0 &{} -f - \zeta &{} 0 \\[10pt] 0 &{} \alpha &{} r_1 &{} r_2 &{} r_3 &{} 0 &{} 0 &{} -\zeta \end{array}\right) \end{aligned}$$

the characteristic equation for the system (13) is obtained as:

$$\begin{aligned} J\left( \textrm{DFE}\right) =&\left( - \beta - m - \zeta - {\lambda }^1\right) \left( \frac{\Pi \left( m p + \omega \left( f + \zeta \right) \right) - \left( \gamma + \upsilon _{1} + \upsilon _{2} + \alpha + \zeta \right) }{\left( \beta + m + \zeta \right) \left( f + \zeta \right) }-{\lambda }^2\right) \left( - r_1 - \delta -\zeta -{\lambda }^3\right) \\ {}&\qquad \left( - r_2 - \zeta - {\lambda }^4\right) \left( - h_2 - r_3 - \delta - \zeta - {\lambda }^5\right) \left( - {\beta }_1 - {\beta }_2 - \zeta -{\lambda }^6\right) \left( - f - \zeta - {\lambda }^7\right) \left( - \zeta - {\lambda }^8\right) , \end{aligned}$$

which implies that

$$\begin{aligned} {\lambda }^1 =&-\left( \beta +m+\zeta \right)< 0, \\ {\lambda }^2 =&\left( \gamma +{\upsilon }_1+{\upsilon }_2+ \omega +\zeta \right) \left( {\mathscr {R}}_0-1\right)<0, \qquad \Longrightarrow \qquad {\mathscr {R}}_0<1. \\ {\lambda }^3 =&-\left( r_1+\delta +\zeta \right)<0, \\ {\lambda }^4 =&-\left( r_2 +\zeta \right)<0, \\ {\lambda }^5 =&- \left( h_2 + r_3 + \delta + \zeta \right)<0, \\ {\lambda }^6 =&-\left( {\beta }_1+{\beta }_2+\zeta \right)<0, \\ {\lambda }^7 =&-\left( f + \zeta \right)<0, \\ {\lambda }^8 =&-\zeta <0. \end{aligned}$$

It is obvious that $\lambda ^{1, 3, 4, 5, 6, 7, 8}$ has negative sign. In addition, we can deduce that $\lambda ^2$ is negative then $\mathscr {R}_0<1$, implying that DFE is asymptotically stable locally. The proof is now complete.

### Sensitivity analysis of ${\mathscr {R}}_0$

The proposed model of COVID-19 presented in system (13) is examined using sensitivity analysis to investigate the effects of the parameters. The sensitivity analysis helps in understanding how changes in each parameter impact the overall dynamics of the model. By varying the parameters within a certain range, researchers can gain insights into which factors have the most significant influence on the spread of the virus. It’s crucial to identify the parameters whose values can be altered relatively easily without significantly altering the model’s dynamics. This information can then be used to inform public health interventions and policies aimed at controlling the spread of COVID-19. Additionally, sensitivity analysis can help researchers identify key areas for further study or data collection to improve the accuracy of the model. Following is a normalized version of the sensitivity indices for the parameters:

15 $$\begin{aligned} \Gamma _{x}= & {} \displaystyle \frac{x}{\mathscr {R}_0} \times \displaystyle \frac{\partial {\mathscr {R}}_0}{\partial x }, \end{aligned}$$

**Table 2:** Sensitivity indices of the $\mathscr {R}_{0}$ against the parameters used in model (13).

Sensitivity index	Value	Sensitivity index	Value
$\Gamma _{m}$	0.8609	$\Gamma _{\upsilon _1}$	$-0.1094$
$\Gamma _{\delta }$	0.0000	$\Gamma _{p}$	0.9992
$\Gamma _{r_2}$	0.0000	$\Gamma _{\omega }$	0.0008
$\Gamma _{\gamma }$	$-0.0604$	$\Gamma _{\beta }$	$-0.0004$
$\Gamma _{r_1}$	0.0000	$\Gamma _{\beta _{2}}$	0.0000
$\Gamma _{\Pi }$	1.0000	$\Gamma _{f}$	$-0.4702$
$\Gamma _{\upsilon _{2}}$	$-0.7335$	$\Gamma _{h_{1}}$	0.0000
$\Gamma _{h_2}$	0.0000	$\Gamma _{r_{3}}$	0.0000
$\Gamma _{\alpha }$	0.0000	$\Gamma _{\beta _{1}}$	$-0.0819$

It is concluded that the reproduction number $\left( \mathscr {R}_0 \right)$ is increasing with $\Pi , \omega , m$ and *p*. This suggests that higher values of $\Pi , \omega , m$ and *p* are associated with a greater potential for disease transmission. Understanding the factors that contribute to an increased reproduction number can help inform public health interventions and control measures to mitigate the spread of infectious diseases. We compute derivatives in order to confirm the sensitivity of the function as follow:

16 $$\begin{aligned} \displaystyle \frac{\partial {\mathscr {R}}_0}{\partial \Pi }= & {} \displaystyle \frac{ p m + \omega \left( f + \zeta \right) }{\left( \gamma +{\upsilon }_1+{\upsilon }_2+ \omega + \zeta \right) \left( \beta + m + \zeta \right) \left( f + \zeta \right) }, \end{aligned}$$

17 $$\begin{aligned} \displaystyle \frac{\partial {\mathscr {R}}_0}{\partial \omega }= & {} \displaystyle \frac{ \Pi }{\left( \gamma +{\upsilon }_1+{\upsilon }_2+ \omega + \zeta \right) \left( \beta + m + \zeta \right) }, \end{aligned}$$

18 $$\begin{aligned} \displaystyle \frac{\partial {\mathscr {R}}_0}{\partial {p}}= & {} \displaystyle \frac{\Pi m}{\left( \gamma +{\upsilon }_1+{\upsilon }_2+ \omega + \zeta \right) \left( \beta + m + \zeta \right) \left( f + \zeta \right) }, \end{aligned}$$

19 $$\begin{aligned} \displaystyle \frac{\partial {\mathscr {R}}_0}{\partial {m}}= & {} \displaystyle \frac{\Pi \left( p \left( \beta + \zeta \right) - \omega \left( f + \zeta \right) \right) }{\left( \gamma +{\upsilon }_1+{\upsilon }_2+ \omega + \zeta \right) \left( \beta + m + \zeta \right) ^{2}\left( f + \zeta \right) }, \end{aligned}$$

**Figure 3:**
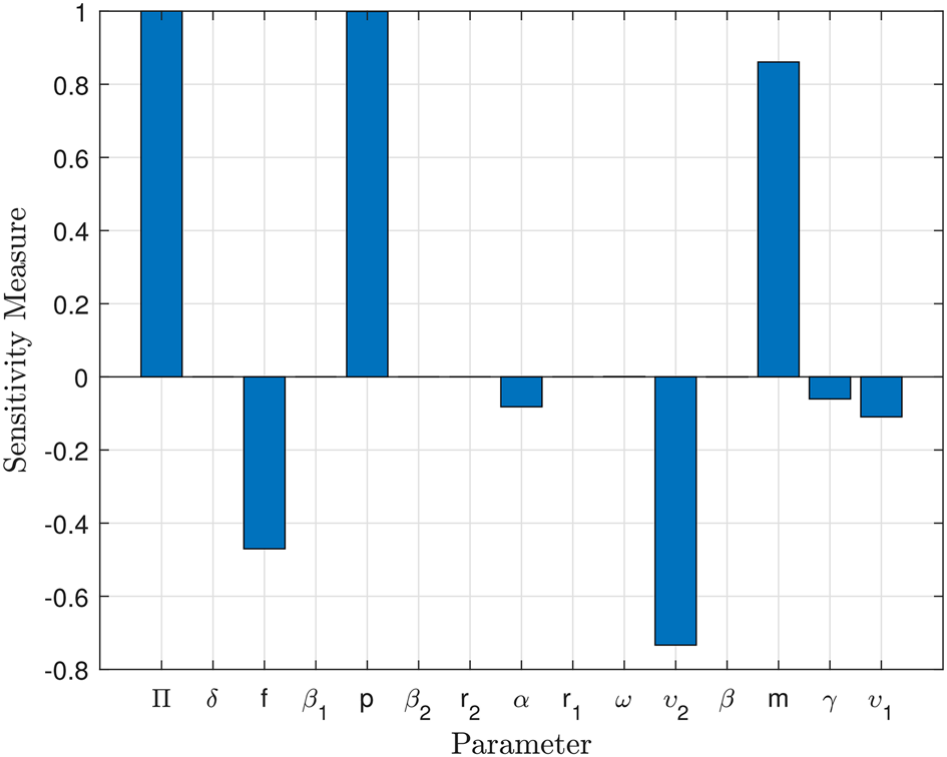
Sensitivity of the basic reproduction number $\mathscr {R}_{0}$ to changes in the model (13) parameters using sensitivity indices.

The sensitivity index being constant based on specific parameters means that it remains unchanged regardless of variations in other factors. On the other hand, a sensitivity index that is free of any independent parameters implies that it is not influenced by any specific variables and can vary independently. Table 2 illustrates the impact of sensitivity indices on the observed results. It provides a clear comparison between the constant sensitivity index and the independent sensitivity index in terms of their effects on different variables. Figure 3 show the partial rank correlation coefficient (PRCC) values for the significance of parameters involved in ${\mathscr {R}}_0$. These PRCC values provide insights into the extent to which each parameter affects the overall value of ${\mathscr {R}}_0$. By analyzing the PRCC values in Fig. 3, researchers can identify the most influential parameters and prioritize them for further investigation or intervention.

**Figure 4:**
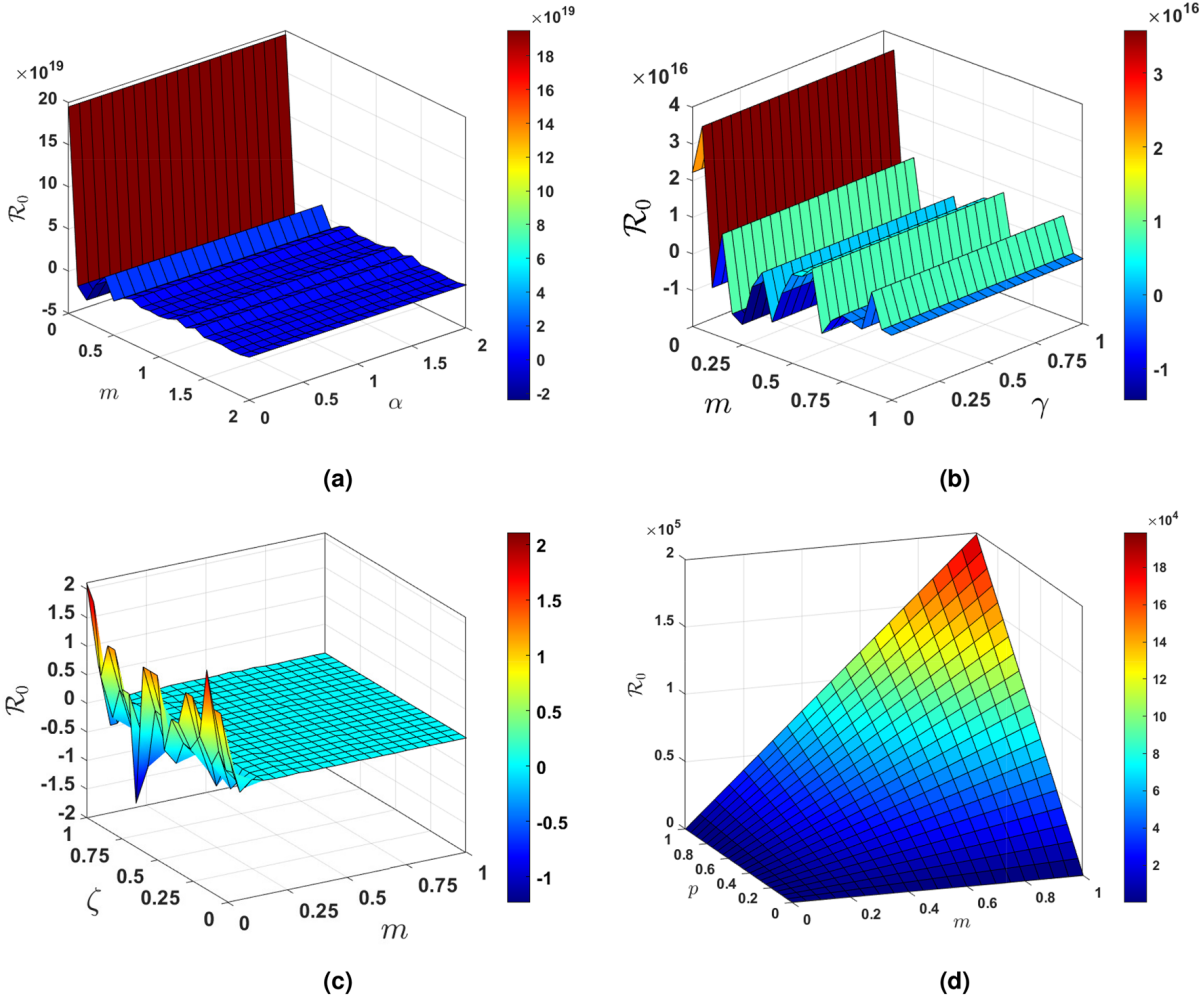
Based on PRCC calculations, this figure shows the clinically importance of parameter involved in $\mathscr {R}_0$ for vaccination parameter *m* in $({\textbf {a}})$ with $\alpha$, $({\textbf {b}})$ with $\gamma$, $({\textbf {c}})$ with $\zeta$ and $({\textbf {d}})$ with *p*.

Figure 4 specifically examines the vaccination parameter *m*, which is a clinically significant parameter utilised in the computation of the basic reproduction number $\mathscr {R}_0$. This parameter plays a crucial role in determining the effectiveness of vaccination campaigns in controlling the spread of infectious diseases. Understanding how changes in *m* impact $\mathscr {R}_0$ is essential for developing successful public health strategies. In various situations, the significance of various parameters is emphasised. For the purpose of figuring out $\mathscr {R}_0$, scenario in Fig. 4a shows what the recovery rate means in terms of the vaccination parameter. Specifically, as the recovery rate increases, the basic reproduction number decreases, indicating that a higher rate of recovery can help reduce the spread of infectious diseases. This highlights the importance of considering multiple parameters when evaluating the impact of vaccination campaigns on disease control. Figure 4b illustrates the clinical significance of the vaccination parameter *m* in conjunction with the rate at which exposed individuals are transferred to quarantine. By analyzing the relationship between *m* and *gamma*, we can better understand how different combinations of vaccination and quarantine strategies can impact disease transmission. This comprehensive approach allows for more effective public health interventions to be developed based on a thorough consideration of various factors influencing disease control. The Scenario in Fig. 4c explores the impact of the natural death rate in relation to the vaccination within the framework of $\mathscr {R}_0$. This analysis helps to determine the optimal balance between vaccination efforts and natural immunity in controlling disease spread. Understanding how the natural death rate influences vaccination strategies can provide valuable insights for public health decision-making. In conclusion, Fig. 4d illustrates how the figure computes $\mathscr {R}_0$ with respect to the vaccination while assessing the clinical significance of the disease exposure rate subsequent to vaccination, referred to as *p*. This information can guide policymakers in making informed decisions about vaccination campaigns and disease control measures. By considering various scenarios and outcomes, public health officials can develop effective strategies to combat the spread of infectious diseases. Each parameter in the mathematical model used to evaluate the effect of vaccination on disease transmission is critical in establishing the basic reproduction number, and these scenarios probably reflect various parts of that model. Understanding the relationship between vaccination and disease transmission is essential for creating successful public health interventions. By analyzing the impact of different parameters, policymakers can tailor strategies to effectively reduce the spread of infectious diseases within communities.

### Existence of endemic equilibrium point

This section examines the possibility of an endemic equilibrium point. Let us use the symbol for the endemic equilibrium point are $\left( S^*, E^*, Q^*, I^*_A, I_S^*, H^*, V^*, R^* \right)$ and endemic equilibrium always satisfies:

20 $$\begin{aligned} \Pi - \omega E^* S^* - \left( \beta + m + \zeta \right) S^*= & {} 0, \nonumber \\ p E^* V^* + \omega S^* E^* - \left( \gamma +{\upsilon }_1+{\upsilon }_2+ \alpha +\zeta \right) E^*= & {} 0, \nonumber \\ \beta S^* + \gamma E^* - \left( \beta _{1} + \beta _{2} + \zeta \right) Q^*= & {} 0, \nonumber \\ \upsilon _{1} E^* + f V^* + \beta _{1} Q^* - \left( r_{1} + \delta +\zeta \right) I^*_A= & {} 0, \nonumber \\ \upsilon _{2} E^* +\beta _{2} Q^* - h_1 H^* I^*_S - \left( r_2 + \zeta \right) I^*_S= & {} 0, \nonumber \\ h_1 H^* I^*_S - \left( h_2 + r_3 + \delta + \zeta \right) H^*= & {} 0, \nonumber \\ m S^* + h_2 H^* - p E^* V^* - \left( f + \zeta \right) V^*= & {} 0, \nonumber \\ \alpha E^* + r_1 I^*_A + r_2 I^*_S + r_3 H^* - \zeta R^*= & {} 0, \end{aligned}$$

As a result, we arrive to the following theorem.

#### Theorem 3.2

*There exist a unique endemic equilibrium point for the system* (10)* and is given as*

21 $$\begin{aligned} S^*= & {} \frac{\Pi X}{\left( \beta + m + \zeta \right) D - \omega p h_1 h_2 \beta \beta _2 \Pi }, \end{aligned}$$

22 $$\begin{aligned} E^*= & {} \frac{1}{X} \left[ \left( \beta + m + \zeta \right) \left( \beta _1 + \beta _2 + \zeta \right) \left( h_2 + r_3 + \delta + \zeta \right) \left( \left( h_1\left( f + \zeta \right) \left( \gamma + \upsilon _1 + \upsilon _2 + \alpha + \zeta \right) \right. \right. \right. \nonumber \\ {}{} & {} \quad \left. \left. \left. - ph_2\left( r_2 + \zeta \right) \right) + \left( mph_1\Pi + \omega h_1\Pi \left( f + \zeta \right) \right) \right) - ph_1h_2\beta _2\beta \Pi \right] , \end{aligned}$$

23 $$\begin{aligned} Q^*= & {} \frac{\beta \Pi X^2 + \gamma F\left( \left( \beta + m + \zeta \right) D - \omega ph_1h_2\beta \beta _2\Pi \right) }{\left( \beta _1 + \beta _2 + \zeta \right) \left( \left( \beta + m + \zeta \right) D - \omega ph_1h_2\beta \beta _2\Pi \right) X}, \end{aligned}$$

24 $$\begin{aligned} I_A^*= & {} \frac{\left( \upsilon _1N + P \right) U + \beta _1T\left( \beta \Pi X + \gamma F\left( \left( \beta + m + \zeta \right) D - \omega ph_1h_2\beta \beta _2\Pi \right) \right) }{\left( r_1 + \delta + \zeta \right) UT},\end{aligned}$$

25 $$\begin{aligned} I_S^*= & {} \frac{h_2 + r_3 + \delta + \zeta }{h_1},\end{aligned}$$

26 $$\begin{aligned} H^*= & {} \frac{h_1\left( \upsilon _2K + M \right) - \left( r_2 + \zeta \right) \left( h_2 + r_3 + \delta + \zeta \right) \left( L\left( h_2 + r_3 + \delta + \zeta \right) \right) }{h_1L\left( h_2 + r_3 + \delta + \zeta \right) }.\end{aligned}$$

27 $$\begin{aligned} V^*= & {} \frac{\left( \gamma + \upsilon _1 + \upsilon _2 + \alpha + \zeta \right) \left( \left( \beta + m + \zeta \right) D - \omega ph_1h_2\beta \beta _2\Pi \right) - \omega \Pi X}{p\left( \left( \beta + m + \zeta \right) D - \omega ph_1h_2\beta \beta _2\Pi \right) }, \end{aligned}$$

28 $$\begin{aligned} R^*= & {} \frac{1}{\zeta LTUX} \left[ \alpha h_1\left( h_2 + r_3 + \delta + \zeta \right) \left( r_1 + \delta + \zeta \right) \left( \left( \beta + m + \zeta \right) \left( \beta _1 + \beta _2 + \zeta \right) \left( h_2 + r_3 + \delta + \zeta \right) \right. \right. \nonumber \\{} & {} \left. \left. \qquad \times \left( \left( h_1\left( f + \zeta \right) \left( \gamma + \upsilon _1 + \upsilon _2 + \alpha + \zeta \right) - ph_2\left( r_2 + \zeta \right) \right) + \left( mph_1\Pi + \omega h_1\Pi \left( f + \zeta \right) \right) \right) \right. \right. \nonumber \\{} & {} \left. \left. \qquad - ph_1h_2\beta _2\beta \Pi \right) LTU + VX \right] . \end{aligned}$$

Here:

$$\begin{aligned} D{} & {} = \left( \left( \beta _1 + \beta _2 + \zeta \right) \left( h_2 + r_3 + \delta + \zeta \right) \left( \omega \left( h_1\left( f + \zeta \right) \left( \gamma + \upsilon _1 + \upsilon _2 + \alpha + \zeta \right) - ph_2\left( r_2 + \zeta \right) \right) + \right. \right. \\ {}{} & {} \quad \left. \left. \left( mph_1\Pi + \omega h_1\Pi \left( f + \zeta \right) \right) - G \right) + ph_2h_1\left( \beta + m + \zeta \right) \left( \upsilon _2\left( \beta _1 + \beta _2 + \zeta \right) + \gamma \beta _2 \right) \right) , \\ F{} & {} = \left( \left( \beta + m + \zeta \right) \left( \beta _1 + \beta _2 + \zeta \right) \left( h_2 + r_3 + \delta + \zeta \right) \left( \left( h_1\left( f + \zeta \right) \left( \gamma + \upsilon _1 + \upsilon _2 + \alpha + \zeta \right) \right. \right. \right. \\ {}{} & {} \quad \left. \left. \left. - ph_2\left( r_2 + \zeta \right) \right) + \left( mph_1\Pi + \omega h_1\Pi \left( f + \zeta \right) \right) \right) - ph_1h_2\beta _2\beta \Pi \right) , \\ G{} & {} = ph_1\left( \beta + m + \zeta \right) \left( \gamma + \upsilon _1 + \upsilon _2 + \alpha + \zeta \right) + \omega \left( h_1\left( f + \zeta \right) \left( \gamma + \upsilon _1 + \upsilon _2 + \alpha + \zeta \right) \right. \\{} & {} \quad \left. - ph_2\left( r_2 + \zeta \right) \right) - ph_1\Pi , \\ K{} & {} = \left( \beta _1 + \beta _2 + \zeta \right) \left( \left( \beta + m + \zeta \right) D - \omega ph_1h_2\beta \beta _2\Pi \right) \left( \left( \beta + m + \zeta \right) \left( \beta _1 + \beta _2 + \zeta \right) \left( h_2 + r_3 \right. \right. \\ {}{} & {} \quad \left. \left. + \delta + \zeta \right) \left( \left( h_1\left( f + \zeta \right) \left( \gamma + \upsilon _1 + \upsilon _2 + \alpha + \zeta \right) - ph_2\left( r_2 + \zeta \right) \right) + \left( mph_1\Pi \right. \right. \right. \\ {}{} & {} \quad \left. \left. \left. + \omega h_1\Pi \left( f + \zeta \right) \right) \right) - ph_1h_2\beta _2\beta \Pi \right) X, \\ L{} & {} = \left( \beta _1 + \beta _2 + \zeta \right) \left( \left( \beta + m + \zeta \right) D - \omega ph_1h_2\beta \beta _2\Pi \right) X^2, \\ M{} & {} = \beta _2\left( \beta \Pi X + \gamma F\left( \left( \beta + m + \zeta \right) D - \omega ph_1h_2\beta \beta _2\Pi \right) \right) X, \\ N{} & {} = \left( \left( \beta + m + \zeta \right) \left( \beta _1 + \beta _2 + \zeta \right) \left( h_2 + r_3 + \delta + \zeta \right) \left( \left( h_1\left( f + \zeta \right) \left( \gamma + \upsilon _1 + \upsilon _2 + \alpha + \zeta \right) \right. \right. \right. \\ {}{} & {} \quad \left. \left. \left. - ph_2\left( r_2 + \zeta \right) \right) + \left( mph_1\Pi + \omega h_1\Pi \left( f + \zeta \right) \right) \right) - ph_1h_2\beta _2\beta \Pi \right) \left( p\left( \left( \beta + m + \zeta \right) D \right. \right. \\ {}{} & {} \quad \left. \left. - \omega ph_1h_2\beta \beta _2\Pi \right) \right) , \\ P{} & {} = \left( f\left( \left( \gamma + \upsilon _1 + \upsilon _2 + \alpha + \zeta \right) \left( \left( \beta + m + \zeta \right) D - \omega ph_1h_2\beta \beta _2\Pi \right) - \omega X \right) \right) X, \\ T{} & {} = p\left( \left( \beta + m + \zeta \right) D - \omega ph_1h_2\beta \beta _2\Pi \right) X, \\ U{} & {} = \left( \beta _1 + \beta _2 + \zeta \right) \left( \left( \beta + m + \zeta \right) D - \omega ph_1h_2\beta \beta _2\Pi \right) X, \\ V{} & {} = r_1h_1L\left( h_2 + r_3 + \delta + \zeta \right) \left( \left( \upsilon _1N + P \right) U + \beta _1T\left( \beta \Pi X + \gamma F\left( \left( \beta + m + \zeta \right) D - \omega ph_1h_2\beta \beta _2\Pi \right) \right) \right) \\ {}{} & {} \quad + \left( r_1 + \delta + \zeta \right) \left( r_2L\left( h_2 + r_3 + \delta + \zeta \right) ^2 + r_3\left( h_1\left( \upsilon _2K + M \right) - \left( r_2 + \zeta \right) \right. \right. \\ {}{} & {} \quad \left. \left. \times \left( h_2 + r_3 + \delta + \zeta \right) ^2L \right) \right) UT, \\ X{} & {} = ph_2h_1\left( \beta + m + \zeta \right) \left( \upsilon _2\left( \beta _1 + \beta _2 + \zeta \right) + \gamma \beta _2 \right) - \left( \beta _1 + \beta _2 + \zeta \right) \left( h_2 + r_3 + \delta + \zeta \right) G. \end{aligned}$$

### Global stability analysis

The following theorem serves as our final statement.

#### Theorem 3.3

*There are no periodic orbit for the system* (10).

#### *Proof*

To acheive this, we turn to Dulac’s criterion. Now, let $X = \left( S, E, I_A, I_S, Q, H, V, R \right)$. Relying on the Dulac’s function

$$\begin{aligned} \mathscr {G} = \frac{1}{SE}, \end{aligned}$$

we acquire

$$\begin{aligned} \displaystyle \mathscr {G}\frac{dS}{dt}= & {} \frac{\Pi }{SE} - \frac{ \beta + m + \zeta }{E} - \omega , \\ \displaystyle \mathscr {G}\frac{dE}{dt}= & {} \frac{p V}{S} - \frac{\gamma + \upsilon _{1} + \upsilon _{2} + \alpha + \zeta }{S} + \omega , \\ \displaystyle \mathscr {G}\frac{dI_A}{dt}= & {} \frac{\upsilon _{1}}{S} + \frac{f V}{SE} + \frac{\beta _{1} Q}{SE} - \frac{\left( r_{1} + \delta +\zeta \right) I_A}{SE}, \\ \displaystyle \mathscr {G}\frac{dI_S}{dt}= & {} \frac{\upsilon _{2}}{S} + \frac{\beta _{2} Q}{SE} - \frac{h_1 H I_S}{SE} - \frac{\left( r_2 + \zeta \right) I_S}{SE}, \\ \displaystyle \mathscr {G}\frac{dQ}{dt}= & {} \frac{\beta }{E} + \frac{\gamma }{S} - \frac{\left( \beta _{1} + \beta _{2} + \zeta \right) Q}{SE}, \\ \displaystyle \mathscr {G}\frac{dH}{dt}= & {} \frac{h_1 H I_S}{SE} - \frac{\left( h_2 + r_3 + \delta + \zeta \right) H}{SE}, \\ \displaystyle \mathscr {G}\frac{dV}{dt}= & {} \frac{m}{E} + \frac{h_2 H}{SE} - \frac{p V}{S} - \frac{\left( f + \delta + \zeta \right) V}{SE}, \\ \displaystyle \mathscr {G}\frac{dR}{dt}= & {} \frac{\alpha }{S} + \frac{r_1 I_A}{SE} + \frac{r_2 I_S}{SE} + \frac{r_3 H}{SE} - \frac{\zeta R}{SE}, \end{aligned}$$

Thus,

$$\begin{aligned} \frac{d\mathscr {G}X}{dt}= & {} \frac{\partial }{\partial S}\left( \mathscr {G}\frac{dS}{dt} \right) + \frac{\partial }{\partial E}\left( \mathscr {G}\frac{dE}{dt} \right) + \frac{\partial }{\partial I_A}\left( \mathscr {G}\frac{dI_A}{dt} \right) + \frac{\partial }{\partial I_S}\left( \mathscr {G}\frac{dI_S}{dt} \right) + \frac{\partial }{\partial Q}\left( \mathscr {G}\frac{dQ}{dt} \right) \\ {}{} & {} + \frac{\partial }{\partial H}\left( \mathscr {G}\frac{dH}{dt} \right) + \frac{\partial }{\partial V}\left( \mathscr {G}\frac{dV}{dt} \right) + \frac{\partial }{\partial R}\left( \mathscr {G}\frac{dR}{dt} \right) , \\ \frac{d\mathscr {G}X}{dt}= & {} \frac{\partial }{\partial S}\left( \frac{\Pi }{SE} - \frac{ \beta + m + \zeta }{E} - \omega \right) + \frac{\partial }{\partial E}\left( \frac{p V}{S} - \frac{\gamma + \upsilon _{1} + \upsilon _{2} + \alpha + \zeta }{S} + \omega \right) \\ {}{} & {} + \frac{\partial }{\partial I_A}\left( \frac{\upsilon _{1}}{S} + \frac{f V}{SE} + \frac{\beta _{1} Q}{SE} - \frac{\left( r_{1} + \delta +\zeta \right) I_A}{SE} \right) + \frac{\partial }{\partial I_S}\left( \frac{\upsilon _{2}}{S} + \frac{\beta _{2} Q}{SE} \right. \\ {}{} & {} \left. - \frac{h_1 H I_S}{SE} - \frac{\left( r_2 + \zeta \right) I_S}{SE} \right) + \frac{\partial }{\partial Q}\left( \frac{\beta }{E} + \frac{\gamma }{S} - \frac{\left( \beta _{1} + \beta _{2} + \zeta \right) Q}{SE} \right) \\ {}{} & {} +\frac{\partial }{\partial H}\left( \frac{h_1 H I_S}{SE} - \frac{\left( h_2 + r_3 + \delta + \zeta \right) H}{SE} \right) + \frac{\partial }{\partial V}\left( \frac{m}{E} + \frac{h_2 H}{SE} - \frac{p V}{S} \right. \\ {}{} & {} \left. - \frac{\left( f + \delta + \zeta \right) V}{SE} \right) + \frac{\partial }{\partial R}\left( \frac{\alpha }{S} + \frac{r_1 I_A}{SE} + \frac{r_2 I_S}{SE} + \frac{r_3 H}{SE} - \frac{\zeta R}{SE} \right) , \\ \frac{d\mathscr {G}X}{dt}= & {} \left( - \frac{\Pi }{S^2 E} \right) - \left( \frac{ r_{1} + \delta +\zeta }{SE} \right) - \left( \frac{h_1 H}{SE} + \frac{r_2 + \zeta }{SE} \right) - \left( \frac{ \beta _{1} + \beta _{2} + \zeta }{SE} \right) \\ {}{} & {} + \left( \frac{h_1 I_S}{SE} - \frac{ h_2 + r_3 + \delta + \zeta }{SE} \right) - \left( \frac{p}{S} + \frac{ f + \delta + \zeta }{SE} \right) - \frac{\zeta }{SE}, \\ \frac{d\mathscr {G}X}{dt}= & {} - \left( \frac{\Pi }{S^2 E} + \frac{ pE + h_1 \left( H - I_S \right) + h_2 + r_1 + r_2 + r_3 + \beta _{1} + \beta _{2} + f + 3 \delta + 6 \zeta }{SE} \right) , \\ \frac{d\mathscr {G}X}{dt}< & {} 0, \end{aligned}$$

The system described by Eq. (10) does not exhibit a periodic orbit. This concludes the proof. $\square$

Since $\mathfrak {B}$ is positively invariant, the Poincar’e-Bendixson theorem states that all solutions to the system (10) begin in $\mathfrak {B}$ and remain there for all *t*. Therefore, it is concluded with the following theorem:

#### Theorem 3.4

*The endemic equilibrium is globally asymptotically stable if* $\mathscr {R}_0 > 1$.

## Mathematical model in fractal-fractional sense

The current study demonstrates that mathematical models created using fractal-fractional operators typically have a higher degree of accuracy and dependability than models created using integer-order operations. With each new formulation, fractal-fractional derivatives have advanced our knowledge of complex systems. As Michel Caputo^24^ first proposed the Caputo fractional derivative, it was a fundamental idea that opened up traditional calculus to include non-local and memory-dependent phenomena. Over time, researchers further refined fractional derivatives to enhance their applicability. Caputo and Fabrizio introduced the Caputo–Fabrizio fractional derivative in 2015 as a revised definition with a smooth kernel, which improved the representation of processes in the real world^25^. Atangana and Baleanu introduces Atangana–Baleanu derivative in 2016 as another variation of the fractional derivative^26^. This derivative was made to better capture the memory effect and non-locality, which means it can be used to model complex systems with interactions that happen over long distances. egarding fractional linear differential equations, Atangana and Qureshi^16^ investigated the Atangana–Baleanu–Caputo fractional derivative in 2020. By integrating fractal elements into the Caputo framework, this variant, which was proposed by Atangana and Baleanu, offered an original viewpoint. Following this, research conducted by Arif et al.^27^ underscored the effectiveness of recently developed fractional fractal derivatives, showcasing their superiority in comparison to conventional fractional derivatives and classical fractional derivatives. The ongoing refinement of mathematical models to enhance comprehension of complex systems across various scientific disciplines^28–33^ is evident in the continuous evolution of these derivatives.

The proposed nonlinear fractional model (10), in the sense of fractal-fractional operatos (discussed in “Preliminaries”), thus has the following structure:

29 $$\begin{aligned} ^{FF}D^{\eta , \tau }_{0,t} \left( S \left( t \right) \right)= & {} \Pi - \omega ES - \left( \beta + m + \zeta \right) S, \nonumber \\ ^{FF}D^{\eta , \tau }_{0,t} \left( E \left( t \right) \right)= & {} p EV + \omega SE - \left( \gamma + \upsilon _{1} + \upsilon _{2} + \alpha + \zeta \right) E, \nonumber \\ ^{FF}D^{\eta , \tau }_{0,t} \left( Q \left( t \right) \right)= & {} \beta S + \gamma E - \left( \beta _{1} + \beta _{2} + \zeta \right) Q, \nonumber \\ ^{FF}D^{\eta , \tau }_{0,t} \left( I_A \left( t \right) \right)= & {} \upsilon _{1} E + f V + \beta _{1} Q - \left( r_{1} + \delta +\zeta \right) I_A, \nonumber \\ ^{FF}D^{\eta , \tau }_{0,t} \left( I_S \left( t \right) \right)= & {} \upsilon _{2} E +\beta _{2} Q - h_1 HI_S - \left( r_2 + \zeta \right) I_S, \nonumber \\ ^{FF}D^{\eta , \tau }_{0,t} \left( H \left( t \right) \right)= & {} h_1 HI_S - \left( h_2 + r_3 + \delta + \zeta \right) H, \nonumber \\ ^{FF}D^{\eta , \tau }_{0,t} \left( V \left( t \right) \right)= & {} m S + h_2 H - p EV - \left( f + \zeta \right) V, \nonumber \\ ^{FF}D^{\eta , \tau }_{0,t} \left( R \left( t \right) \right)= & {} \alpha E + r_1 I_A + r_2 I_S + r_3 H - \zeta R, \end{aligned}$$

under the assumption that the variables are all positive, and with appropriate initial conditions $^{FF}D^{\eta , \tau }_{0,t} \left( \cdot \right)$ is the fractal-fractional derivative of order $\eta$ with fractal dimension $\tau$.

### Existence and uniqueness results for the solution

Here, we generalize the presented model to a fractal-fractional order and use the fixed point theorem to check whether or not this new model has unique solutions. This generalization is motivated by the aforementioned application. Let us define the kernals to demonstrate the existence of a unique solution under Atangana–Baleanu fractal-fractional derivative

$$\begin{aligned} F_1 \left( t, S \left( t \right) \right)= & {} \Pi - \omega ES - \left( \beta + m + \zeta \right) S, \\ F_2 \left( t, E \left( t \right) \right)= & {} p EV + \omega SE - \left( \gamma + \upsilon _{1} + \upsilon _{2} + \alpha + \zeta \right) E, \\ F_3 \left( t, Q \left( t \right) \right)= & {} \beta S + \gamma E - \left( \beta _{1} + \beta _{2} + \zeta \right) Q, \\ F_4 \left( t, I_A \left( t \right) \right)= & {} \upsilon _{1} E + f V + \beta _{1} Q - \left( r_{1} + \delta +\zeta \right) I_A,\\ F_5 \left( t, I_S \left( t \right) \right)= & {} \upsilon _{2} E +\beta _{2} Q - h_1 HI_S - \left( r_2 + \zeta \right) I_S, \\ F_6 \left( t, H \left( t \right) \right)= & {} h_1 HI_S - \left( h_2 + r_3 + \delta + \zeta \right) H, \\ F_7 \left( t, V \left( t \right) \right)= & {} m S + h_2 H - p EV - \left( f + \zeta \right) V, \\ F_8 \left( t, R \left( t \right) \right)= & {} \alpha E + r_1 I_A + r_2 I_S + r_3 H - \zeta R, \end{aligned}$$

where $F_1 \left( t, S \left( t \right) \right) , F_2 \left( t, E \left( t \right) \right) , F_3 \left( t, Q \left( t \right) \right) , F_4 \left( t, I_A \left( t \right) \right) , F_5 \left( t, I_S \left( t \right) \right) , F_6 \left( t, H \left( t \right) \right) , F_7 \left( t, V \left( t \right) \right)$ and $F_8 \left( t, R \left( t \right) \right)$ are contraction with respect to $S, E, Q, I_{A}, I_{S}, H, V$ and *R* respectively. Here we consider a model (29) with fractal-fractional derivative for ordinary differential equations given as in the Atanagana–Baleanu case.

30 $$\begin{aligned} ^{FFABC}D^{\eta }_{0,t} \left( W \left( t \right) \right)= & {} F \left( t, W \left( t \right) \right) , \end{aligned}$$

where

$$\begin{aligned} W \left( t \right) = \left( \begin{array}{c} S \left( t \right) \\ E \left( t \right) \\ I_A \left( t \right) \\ I_S\left( t \right) \\ Q\left( t \right) \\ H\left( t \right) \\ V \left( t \right) \\ R \left( t \right) \end{array} \right) , \qquad \qquad W \left( 0 \right) = \left( \begin{array}{c} S \left( 0 \right) = S_0 \\ E \left( 0 \right) = E_0 \\ I_A \left( 0 \right) = I_{A_0} \\ I_S\left( 0 \right) = I_{S_0} \\ Q\left( 0 \right) = Q_0 \\ H\left( 0 \right) = H_0 \\ V \left( 0 \right) = V_0 \\ R \left( 0 \right) = R_0 \end{array} \right) = W_0, \qquad \qquad F \left( t, W \left( t \right) \right) = \left( \begin{array}{c} F_1 \left( t, S \left( t \right) \right) \\ F_2 \left( t, E \left( t \right) \right) \\ F_3 \left( t, I_A \left( t \right) \right) \\ F_4 \left( t, I_S \left( t \right) \right) \\ F_5 \left( t, Q \left( t \right) \right) \\ F_6 \left( t, H \left( t \right) \right) \\ F_7 \left( t, V \left( t \right) \right) \\ F_8 \left( t, R \left( t \right) \right) \end{array} \right) , \end{aligned}$$

Then using the fact that $^{FFABC}D^{\eta }_{0,t} \left( W \left( t \right) \right)$ can be written as:

31 $$\begin{aligned}= & {} \frac{AB \left( \eta \right) }{1 - \eta } \frac{d}{dt^\tau } \int _{0}^{t} F \left( \mu , W \left( \mu \right) \right) E_\eta \left( - \frac{\eta }{1 - \eta } \left( t - \tau \right) ^{\eta } \right) d\tau , \end{aligned}$$

Consequently, we can change the integral to because it is differentiable

32 $$\begin{aligned}= & {} \frac{1}{\tau t^{\tau - 1}} \frac{AB \left( \eta \right) }{1 - \eta } \frac{d}{dt^\tau } \int _{0}^{t} F \left( \mu , W \left( \mu \right) \right) E_\eta \left( - \frac{\eta }{1 - \eta } \left( t - \tau \right) ^{\eta } \right) d\tau , \end{aligned}$$

Therefore, Eq. (30) can be expressed as follows:

33 $$\begin{aligned} \frac{AB \left( \eta \right) }{1 - \eta } \frac{d}{dt^\tau } \int _{0}^{t} F \left( \mu , W \left( \mu \right) \right) E_\eta \left( - \frac{\eta }{1 - \eta } \left( t - \tau \right) ^{\eta } \right) d\tau= & {} \tau t^{\tau - 1} F \left( t, W \left( t \right) \right) , \end{aligned}$$

After applying the integral, we get the right hand side by replacing it with the Caputo

34 $$\begin{aligned} W \left( t \right) - W \left( t_0 \right)= & {} \frac{1 - \eta }{AB \left( \eta \right) } \tau t^{\tau - 1} F \left( t, W \left( t \right) \right) + \frac{\eta \tau }{AB \left( \eta \right) \Gamma \left( \eta \right) } \int _{0}^{t} \left( t - \tau \right) ^{\eta - 1} F \left( \mu , W \left( \mu \right) \right) \mu ^{\tau - 1} d\tau , \end{aligned}$$

Let $W \left( t_0 \right) = W \left( 0 \right)$ ; then the Picard iteration is defined by

35 $$\begin{aligned} W_{k+1} \left( t \right)= & {} \frac{1 - \eta }{AB \left( \eta \right) } \tau t^{\tau - 1} F \left( t, W_k \left( t \right) \right) + \frac{\eta \tau }{AB \left( \eta \right) \Gamma \left( \eta \right) } \int _{0}^{t} \left( t - \tau \right) ^{\eta - 1} F \left( \mu , W_k \left( \mu \right) \right) \mu ^{\tau - 1} d\tau , \end{aligned}$$

There exist an upper bond for the non-linear functions $S\left( t \right) , E\left( t \right) , Q\left( t \right) , I_A\left( t \right) , I_S\left( t \right) , H\left( t \right) , V\left( t \right)$ and $R\left( t \right)$ if and only if Lipschitz condition are satisfies by the kernels $F_1 \left( t, S \left( t \right) \right) ,\, F_2 \left( t, E \left( t \right) \right) ,\, F_3 \left( t, Q \left( t \right) \right) ,\, F_4 \left( t, I_A \left( t \right) \right) ,\, F_5 \left( t, I_S \left( t \right) \right) ,\, F_6 \left( t, H \left( t \right) \right) ,\, F_7 \left( t, V \left( t \right) \right)$ and $F_8 \left( t, R \left( t \right) \right)$ for $0 \le M_i < 1, i = 1, 2, 3, \dots , 8.$ Indeed, suppose $S_1\left( t \right)$ and $S_2\left( t \right)$ be two functions, then we get

36 $$\begin{aligned} \left\| F_1\left( t,S_1\right) -F_1\left( t,S_2\right) \right\|= & {} \left\| \left( \Pi -\omega E S_1 - \left( \beta +m+\zeta \right) S_1\right) -\left( \Pi - \omega E S_2 - \left( \beta +m+\zeta \right) S_2\right) \right\| , \nonumber \\= & {} \left\| \left( \omega E+\beta +m+\zeta \right) S_1-\left( \omega E+\beta +m+\zeta \right) S_2\right\| , \nonumber \\\le & {} \left( \omega \sup _{t \in \left[ 0, T \right] }|E| +\beta +m+\zeta \right) \left\| S_1-S_2\right\| , \nonumber \\\le & {} M_1\left\| S_1-S_2\right\| , \end{aligned}$$

where $$ M_1 = \left( \omega \sup _{t \in \left[ 0, T \right] }|E| +\beta +m+\zeta \right) $$, thus

37 $$\begin{aligned} \left\| F_1\left( t,S_1\right) -F_1\left( t,S_2\right) \right\|\le & {} M_1\left\| S_1-S_2\right\| . \end{aligned}$$

By following the identical technique as described in Eq. (36) previously mentioned, we obtain

$$\begin{aligned} \left\| F_2\left( t,E_1\right) -F_2\left( t,E_2\right) \right\|\le & {} M_2 \left\| E_1 - E_2\right\| , \\ \left\| F_3\left( t,Q_1\right) -F_3\left( t,Q_2\right) \right\|\le & {} M_3\left\| Q_1-Q_2\right\| , \\ \left\| F_4\left( t,I_{A_{1}}\right) -F_4\left( t,I_{A_{2}}\right) \right\|\le & {} M_4\left\| I_{A_{1}}-I_{A_{2}}\right\| , \\ \left\| F_5\left( t,I_{S_{1}}\right) -F_5\left( t,I_{S_{2}}\right) \right\|\le & {} M_5\left\| I_{S_{1}}-I_{S_{2}}\right\| , \\ \left\| F_6\left( t,H_1\right) -F_6\left( t,H_2\right) \right\|\le & {} M_6 \left\| H_1 - H_2 \right\| , \\ \left\| F_7\left( t,V_1\right) -F_7\left( 
t,V_2\right) \right\|\le & {} M_7\left\| V_1-V_2\right\| , \\ \left\| F_8\left( t,R_1\right) -F_8\left( t,R_2\right) \right\|\le & {} M_8\left\| R_1-R_2\right\| , \end{aligned}$$

where the equivalent Lipschitz constants for the functions $F_{i} \left( \cdot \right)$ are $M_i$ for $\left( i = 1, 2, 3, \dots , 8 \right)$. Then the Picard operator is defined as

38 $$\begin{aligned} \Lambda \left( W \left( t \right) \right)= & {} W_0 + \frac{1 - \eta }{AB \left( \eta \right) } \tau t^{\tau - 1} F \left( t, W \left( t \right) \right) + \frac{\eta \tau }{AB \left( \eta \right) \Gamma \left( \eta \right) } \int _{0}^{t} \left( t - \tau \right) ^{\eta - 1} F \left( \mu , W \left( \mu \right) \right) \mu ^{\tau - 1} d\tau , \end{aligned}$$

It is worth mentioning that the solution of the fractional model (29) is bounded. In addition, since $F_1 \left( t, S \left( t \right) \right) , F_2 \left( t, E \left( t \right) \right) , F_3 \left( t, Q \left( t \right) \right) ,$ $F_4 \left( t, I_A \left( t \right) \right) , F_5 \left( t, I_S \left( t \right) \right) , F_6 \left( t, H \left( t \right) \right) , F_7 \left( t, V \left( t \right) \right)$ and $F_8 \left( t, R \left( t \right) \right)$ are contraction, we can write

$$\begin{aligned} \left\| \Lambda \left( t,W_1 \left( t \right) \right) - \Lambda \left( t, W_2 \left( t \right) \right) \right\|\le & {} M \left\| W_1 \left( t \right) - W_2 \left( t \right) \right\| \end{aligned}$$

where $M < 1$. Also, by using Eq. (38), we get

39 $$\begin{aligned} \left\| W \left( t \right) - W \left( 0 \right) \right\|= & {} \left\| \frac{1 - \eta }{AB \left( \eta \right) } \tau t^{\tau - 1} F \left( t, W \left( t \right) \right) + \frac{\eta \tau }{AB \left( \eta \right) \Gamma \left( \eta \right) } \int _{0}^{t} \left( t - \tau \right) ^{\eta - 1} F \left( \mu , W \left( \mu \right) \right) \mu ^{\tau - 1} d\tau \right\| , \nonumber \\\le & {} \frac{1 - \eta }{AB \left( \eta \right) } \tau t^{\tau - 1} \left\| F \left( t, W \left( t \right) \right) \right\| + \frac{\eta \tau }{AB \left( \eta \right) \Gamma \left( \eta \right) } \int _{0}^{t} \left( t - \tau \right) ^{\eta - 1} \left\| F \left( \mu , W \left( \mu \right) \right) \right\| \mu ^{\tau - 1} d\tau , \nonumber \\\le & {} \left( \frac{1 - \eta }{AB \left( \eta \right) } \tau t^{\tau - 1} + \frac{\eta \tau t^\eta }{AB \left( \eta \right) \Gamma \left( \eta \right) } \right) M \le K M, \end{aligned}$$

where $KM < 1$. Now, by using the definition of the Picard operator (38), we provide

40 $$\begin{aligned} \left\| W_1 \left( t \right) - W_2 \left( t \right) \right\|= & {} \left\| \frac{1 - \eta }{AB \left( \eta \right) } \tau t^{\tau - 1} F \left( t, W \left( t \right) \right) + \frac{\eta \tau }{AB \left( \eta \right) \Gamma \left( \eta \right) } \int _{0}^{t} \left( t - \tau \right) ^{\eta - 1} F \left( \mu , W \left( \mu \right) \right) \mu ^{\tau - 1} d\tau \right\| , \nonumber \\\le & {} \frac{1 - \eta }{AB \left( \eta \right) } \tau t^{\tau - 1} \left\| F \left( t, W \left( t \right) \right) \right\| + \frac{\eta \tau }{AB \left( \eta \right) \Gamma \left( \eta \right) } \int _{0}^{t} \left( t - \tau \right) ^{\eta - 1} \left\| F \left( \mu , W \left( \mu \right) \right) \right\| \mu ^{\tau - 1} d\tau , \nonumber \\\le & {} \left( \frac{1 - \eta }{AB \left( \eta \right) } \tau t^{\tau - 1} + \frac{\eta \tau t^\eta }{AB \left( \eta \right) \Gamma \left( \eta \right) } \right) M \le K M, \end{aligned}$$

## Numerical scheme with fractal-fractional operators

Three numerical methods for the Caputo, the Caputo–Fabrizio, and the Atangana–Baleanu with fractal-fractional operators are presented here. These numerical methods are used to solve fractional differential equations. They provide efficient and accurate approximations for the solutions of such equations, which often arise in various scientific and engineering applications.

### Fractal fractional derivative in Caputo sense

In this section, the fractal fractional model (29) is discussed with fractal fractional in the sense of Caputo as that of the differentiation operator. We present the concept of fractal dimension as a means of quantifying self-similar patterns within the mathematical model described in the system (29). Given that the fractional integral exhibits differentiability in the aforementioned equations, it is possible to modify them to accommodate the Volterra type scenario. This results in the Riemann-Liouville interpretation of the fractal-fractional derivative being written as

41 $$\begin{aligned} \frac{1}{\Gamma \left( 1 - \eta \right) } \frac{d}{dt} \int _{0}^{t} \left( t - \tau \right) ^{\eta } f \left( \tau \right) d \tau \frac{1}{\tau t^{\tau - 1}}, \end{aligned}$$

such that system (29) becomes

$$\begin{aligned} ^{RL}D^{\eta }_{0,t} \left( S \left( t \right) \right)= & {} \tau t^{\tau - 1} \left[ \Pi - \omega ES - \left( \beta + m + \zeta \right) S\right] , \\ ^{RL}D^{\eta }_{0,t} \left( E \left( t \right) \right)= & {} \tau t^{\tau - 1} \left[ p EV + \omega SE - \left( \gamma + \upsilon _{1} + \upsilon _{2} + \alpha + \zeta \right) E\right] , \\ ^{RL}D^{\eta }_{0,t} \left( Q \left( t \right) \right)= & {} \tau t^{\tau - 1} \left[ \beta S + \gamma E - \left( \beta _{1} + \beta _{2} + \zeta \right) Q\right] , \\ ^{RL}D^{\eta }_{0,t} \left( I_A \left( t \right) \right)= & {} \tau t^{\tau - 1} \left[ \upsilon _{1} E + f V + \beta _{1} Q - \left( r_{1} + \delta +\zeta \right) I_A\right] , \\ ^{RL}D^{\eta }_{0,t} \left( I_S \left( t \right) \right)= & {} \tau t^{\tau - 1} \left[ \upsilon _{2} E +\beta _{2} Q - h_1 HI_S - \left( r_2 + \zeta \right) I_S\right] , \\ ^{RL}D^{\eta }_{0,t} \left( H \left( t \right) \right)= & {} \tau t^{\tau - 1} \left[ h_1 HI_S - \left( h_2 + r_3 + \delta + \zeta \right) H\right] , \\ ^{RL}D^{\eta }_{0,t} \left( V \left( t \right) \right)= & {} \tau t^{\tau - 1} \left[ m S + h_2 H - p EV - \left( f + \zeta \right) V\right] , \\ ^{RL}D^{\eta }_{0,t} \left( R \left( t \right) \right)= & {} \tau t^{\tau - 1} \left[ \alpha E + r_1 I_A + r_2 I_S + r_3 H - \zeta R\right] , \end{aligned}$$

The Riemann–Liouville derivative is now replaced with the Caputo derivative in order to apply the integer-order initial conditions. The results shown below are then obtained by applying the Riemann–Liouville fractional integral to both sides:

$$\begin{aligned} S \left( t \right)= & {} S \left( 0 \right) + \frac{\tau }{\Gamma \left( \eta \right) } \int _{0}^{t} \mu ^{\tau - 1} \left( t - \mu \right) ^{\eta - 1} F_1 \left( \mu , S \left( \mu \right) \right) d \mu , \nonumber \\ E \left( t \right)= & {} E \left( 0 \right) + \frac{\tau }{\Gamma \left( \eta \right) } \int _{0}^{t} \mu ^{\tau - 1} \left( t - \mu \right) ^{\eta - 1} F_2 \left( \mu , E \left( \mu \right) \right) d \mu , \nonumber \\ Q \left( t \right)= & {} Q \left( 0 \right) + \frac{\tau }{\Gamma \left( \eta \right) } \int _{0}^{t} \mu ^{\tau - 1} \left( t - \mu \right) ^{\eta - 1} F_3 \left( \mu , Q \left( \mu \right) \right) d \mu , \nonumber \\ I_A \left( t \right)= & {} I_A \left( 0 \right) + \frac{\tau }{\Gamma \left( \eta \right) } \int _{0}^{t} \mu ^{\tau - 1} \left( t - \mu \right) ^{\eta - 1} F_4 \left( \mu , I_A \left( \mu \right) \right) d \mu , \nonumber \\ I_S \left( t \right)= & {} I_S \left( 0 \right) + \frac{\tau }{\Gamma \left( \eta \right) } \int _{0}^{t} \mu ^{\tau - 1} \left( t - \mu \right) ^{\eta - 1} F_5 \left( \mu , I_S \left( \mu \right) \right) d \mu , \nonumber \\ H \left( t \right)= & {} H \left( 0 \right) + \frac{\tau }{\Gamma \left( \eta \right) } \int _{0}^{t} \mu ^{\tau - 1} \left( t - \mu \right) ^{\eta - 1} F_6 \left( \mu , H \left( \mu \right) \right) d \mu , \nonumber \\ V \left( t \right)= & {} V \left( 0 \right) + \frac{\tau }{\Gamma \left( \eta \right) } \int _{0}^{t} \mu ^{\tau - 1} \left( t - \mu \right) ^{\eta - 1} F_7 \left( \mu , V \left( \mu \right) \right) d \mu , \nonumber \\ R \left( t \right)= & {} R \left( 0 \right) + \frac{\tau }{\Gamma \left( \eta \right) } \int _{0}^{t} \mu ^{\tau - 1} \left( t - \mu \right) ^{\eta - 1} F_8 \left( \mu , R \left( \mu \right) \right) d \mu , \end{aligned}$$

At $t_{n + 1}$, we now present the numerical scheme for this system, which makes use of an original technique. The system undergoes a transformation.

$$\begin{aligned} S \left( t_{n + 1} \right)= & {} S \left( 0 \right) + \frac{\tau }{\Gamma \left( \eta \right) } \int _{0}^{t_{n+1}} \mu ^{\tau - 1} \left( t_{n + 1} - \mu \right) ^{\eta - 1} F_1 \left( \mu , S \left( \mu \right) \right) d \mu , \\ E \left( t_{n + 1} \right)= & {} E \left( 0 \right) + \frac{\tau }{\Gamma \left( \eta \right) } \int _{0}^{t_{n+1}} \mu ^{\tau - 1} \left( t_{n + 1} - \mu \right) ^{\eta - 1} F_2 \left( \mu , E \left( \mu \right) \right) d \mu , \\ Q \left( t_{n + 1} \right)= & {} Q \left( 0 \right) + \frac{\tau }{\Gamma \left( \eta \right) } \int _{0}^{t_{n+1}} \mu ^{\tau - 1} \left( t_{n + 1} - \mu \right) ^{\eta - 1} F_3 \left( \mu , Q \left( \mu \right) \right) d \mu , \\ I_A \left( t_{n + 1} \right)= & {} I_A \left( 0 \right) + \frac{\tau }{\Gamma \left( \eta \right) } \int _{0}^{t_{n+1}} \mu ^{\tau - 1} \left( t_{n + 1} - \mu \right) ^{\eta - 1} F_4 \left( \mu , I_A \left( \mu \right) \right) d \mu , \\ I_S \left( t_{n + 1} \right)= & {} I_S \left( 0 \right) + \frac{\tau }{\Gamma \left( \eta \right) } \int _{0}^{t_{n+1}} \mu ^{\tau - 1} \left( t_{n + 1} - \mu \right) ^{\eta - 1} F_5 \left( \mu , I_S \left( \mu \right) \right) d \mu , \\ H \left( t_{n + 1} \right)= & {} H \left( 0 \right) + \frac{\tau }{\Gamma \left( \eta \right) } \int _{0}^{t_{n+1}} \mu ^{\tau - 1} \left( t_{n + 1} - \mu \right) ^{\eta - 1} F_6 \left( \mu , H \left( \mu \right) \right) d \mu , \\ V \left( t_{n + 1} \right)= & {} V \left( 0 \right) + \frac{\tau }{\Gamma \left( \eta \right) } \int _{0}^{t_{n+1}} \mu ^{\tau - 1} \left( t_{n + 1} - \mu \right) ^{\eta - 1} F_7 \left( \mu , V \left( \mu \right) \right) d \mu , \\ R \left( t_{n + 1} \right)= & {} R \left( 0 \right) + \frac{\tau }{\Gamma \left( \eta \right) } \int _{0}^{t_{n+1}} \mu ^{\tau - 1} \left( t_{n + 1} - \mu \right) ^{\eta - 1} F_8 \left( \mu , R \left( \mu \right) \right) d \mu , \end{aligned}$$

Then we approximate the above obtained integrals to

$$\begin{aligned} S \left( t_{n + 1} \right)= & {} S \left( 0 \right) + \frac{\tau }{\Gamma \left( \eta \right) } \sum _{k = 0}^{n} \int _{t_k}^{t_{k+1}} \mu ^{\tau - 1} \left( t_{n + 1} - \mu \right) ^{\eta - 1} F_1 \left( \mu , S \left( \mu \right) \right) d \mu , \\ E \left( t_{n + 1} \right)= & {} E \left( 0 \right) + \frac{\tau }{\Gamma \left( \eta \right) } \sum _{k = 0}^{n} \int _{t_k}^{t_{k+1}} \mu ^{\tau - 1} \left( t_{n + 1} - \mu \right) ^{\eta - 1} F_2 \left( \mu , E \left( \mu \right) \right) d \mu , \\ Q \left( t_{n + 1} \right)= & {} Q \left( 0 \right) + \frac{\tau }{\Gamma \left( \eta \right) } \sum _{k = 0}^{n} \int _{t_k}^{t_{k+1}} \mu ^{\tau - 1} \left( t_{n + 1} - \mu \right) ^{\eta - 1} F_3 \left( \mu , Q \left( \mu \right) \right) d \mu , \\ I_A \left( t_{n + 1} \right)= & {} I_A \left( 0 \right) + \frac{\tau }{\Gamma \left( \eta \right) } \sum _{k = 0}^{n} \int _{t_k}^{t_{k+1}} \mu ^{\tau - 1} \left( t_{n + 1} - \mu \right) ^{\eta - 1} F_4 \left( \mu , I_A \left( \mu \right) \right) d \mu , \\ I_S \left( t_{n + 1} \right)= & {} I_S \left( 0 \right) + \frac{\tau }{\Gamma \left( \eta \right) } \sum _{k = 0}^{n} \int _{t_k}^{t_{k+1}} \mu ^{\tau - 1} \left( t_{n + 1} - \mu \right) ^{\eta - 1} F_5 \left( \mu , I_S \left( \mu \right) \right) d \mu , \\ H \left( t_{n + 1} \right)= & {} H \left( 0 \right) + \frac{\tau }{\Gamma \left( \eta \right) } \sum _{k = 0}^{n} \int _{t_k}^{t_{k+1}} \mu ^{\tau - 1} \left( t_{n + 1} - \mu \right) ^{\eta - 1} F_6 \left( \mu , H \left( \mu \right) \right) d \mu , \\ V \left( t_{n + 1} \right)= & {} V \left( 0 \right) + \frac{\tau }{\Gamma \left( \eta \right) } \sum _{k = 0}^{n} \int _{t_k}^{t_{k+1}} \mu ^{\tau - 1} \left( t_{n + 1} - \mu \right) ^{\eta - 1} F_7 \left( \mu , V \left( \mu \right) \right) d \mu , \\ R \left( t_{n + 1} \right)= & {} R \left( 0 \right) + \frac{\tau }{\Gamma \left( \eta \right) } \sum _{k = 0}^{n} \int _{t_k}^{t_{k+1}} \mu ^{\tau - 1} \left( t_{n + 1} - \mu \right) ^{\eta - 1} F_8 \left( \mu , R \left( \mu \right) \right) d \mu , \end{aligned}$$

Using Lagrangian piece-wise interpolation, we make the function approximations $\mu ^{\tau - 1} F_{i} \left( \mu , W \left( \mu \right) \right)$ for $i = 1, 2, 3, 4, 5, 6, 7, 8$ and $W = \left[ S, E, Q, I_A, I_S, H, V, R \right] ^{T}$ within the finite interval $\left[ t_{j}, t_{j +1}\right]$ is now written as:

$$\begin{aligned} P^1_j \left( \mu \right)= & {} \frac{\mu - t_{j-1}}{t_j - t_{j-1}} t^{\tau - 1}_j F_1 \left( t_j, S \left( t_j \right) \right) - \frac{\mu - t_{j}}{t_j - t_{j-1}} t^{\tau - 1}_{j-1} F_1 \left( t_{j-1}, S \left( t_{j-1} \right) \right) , \\ P^2_j \left( \mu \right)= & {} \frac{\mu - t_{j-1}}{t_j - t_{j-1}} 
t^{\tau - 1}_j F_2 \left( t_j, E \left( t_j \right) \right) - \frac{\mu - t_{j}}{t_j - t_{j-1}} t^{\tau - 1}_{j-1} F_2 \left( t_{j-1}, E \left( t_{j-1} \right) \right) , \\ P^3_j \left( \mu \right)= & {} \frac{\mu - t_{j-1}}{t_j - t_{j-1}} t^{\tau - 1}_j F_3 \left( t_j, Q \left( t_j \right) \right) - \frac{\mu - t_{j}}{t_j - t_{j-1}} t^{\tau - 1}_{j-1} F_3 \left( t_{j-1}, Q \left( t_{j-1} \right) \right) , \\ P^4_j \left( \mu \right)= & {} \frac{\mu - t_{j-1}}{t_j - t_{j-1}} t^{\tau - 1}_j F_4 \left( t_j, I_A \left( t_j \right) \right) - \frac{\mu - t_{j}}{t_j - t_{j-1}} t^{\tau - 1}_{j-1} F_4 \left( t_{j-1}, I_A \left( t_{j-1} \right) \right) , \\ P^5_j \left( \mu \right)= & {} \frac{\mu - t_{j-1}}{t_j - t_{j-1}} t^{\tau - 1}_j F_5 \left( t_j, I_S \left( t_j \right) \right) - \frac{\mu - t_{j}}{t_j - t_{j-1}} t^{\tau - 1}_{j-1} F_5 \left( t_{j-1}, I_S \left( t_{j-1} \right) \right) , \\ P^6_j \left( \mu \right)= & {} \frac{\mu - t_{j-1}}{t_j - t_{j-1}} t^{\tau - 1}_j F_6 \left( t_j, H \left( t_j \right) \right) - \frac{\mu - t_{j}}{t_j - t_{j-1}} t^{\tau - 1}_{j-1} F_6 \left( t_{j-1}, H \left( t_{j-1} \right) \right) , \\ P^7_j \left( \mu \right)= & {} \frac{\mu - t_{j-1}}{t_j - t_{j-1}} t^{\tau - 1}_j F_7 \left( t_j, V \left( t_j \right) \right) - \frac{\mu - t_{j}}{t_j - t_{j-1}} t^{\tau - 1}_{j-1} F_7 \left( t_{j-1}, V \left( t_{j-1} \right) \right) , \\ P^8_j \left( \mu \right)= & {} \frac{\mu - t_{j-1}}{t_j - t_{j-1}} t^{\tau - 1}_j F_8 \left( t_j, R \left( t_j \right) \right) - \frac{\mu - t_{j}}{t_j - t_{j-1}} t^{\tau - 1}_{j-1} F_8 \left( t_{j-1}, R \left( t_{j-1} \right) \right) , \end{aligned}$$

Thus, we obtain

$$\begin{aligned} S \left( t_{n + 1} \right)= & {} S \left( 0 \right) + \frac{\tau }{\Gamma \left( \eta \right) } \sum _{k = 0}^{n} \int _{t_k}^{t_{k+1}} \mu ^{\tau - 1} \left( t_{n + 1} - \mu \right) ^{\eta - 1} P^1_k \left( \mu \right) d \mu , \\ E \left( t_{n + 1} \right)= & {} E \left( 0 \right) + \frac{\tau }{\Gamma \left( \eta \right) } \sum _{k = 0}^{n} \int _{t_k}^{t_{k+1}} \mu ^{\tau - 1} \left( t_{n + 1} - \mu \right) ^{\eta - 1} P^2_k \left( \mu \right) d \mu , \\ Q \left( t_{n + 1} \right)= & {} Q \left( 0 \right) + \frac{\tau }{\Gamma \left( \eta \right) } \sum _{k = 0}^{n} \int _{t_k}^{t_{k+1}} \mu ^{\tau - 1} \left( t_{n + 1} - \mu \right) ^{\eta - 1} P^3_k \left( \mu \right) d \mu , \\ I_A \left( t_{n + 1} \right)= & {} I_A \left( 0 \right) + \frac{\tau }{\Gamma \left( \eta \right) } \sum _{k = 0}^{n} \int _{t_k}^{t_{k+1}} \mu ^{\tau - 1} \left( t_{n + 1} - \mu \right) ^{\eta - 1} P^4_k \left( \mu \right) d \mu , \\ I_S \left( t_{n + 1} \right)= & {} I_S \left( 0 \right) + \frac{\tau }{\Gamma \left( \eta \right) } \sum _{k = 0}^{n} \int _{t_k}^{t_{k+1}} \mu ^{\tau - 1} \left( t_{n + 1} - \mu \right) ^{\eta - 1} P^5_k \left( \mu \right) d \mu , \\ H \left( t_{n + 1} \right)= & {} H \left( 0 \right) + \frac{\tau }{\Gamma \left( \eta \right) } \sum _{k = 0}^{n} \int _{t_k}^{t_{k+1}} \mu ^{\tau - 1} \left( t_{n + 1} - \mu \right) ^{\eta - 1} P^6_k \left( \mu \right) d \mu , \\ V \left( t_{n + 1} \right)= & {} V \left( 0 \right) + \frac{\tau }{\Gamma \left( \eta \right) } \sum _{k = 0}^{n} \int _{t_k}^{t_{k+1}} \mu ^{\tau - 1} \left( t_{n + 1} - \mu \right) ^{\eta - 1} P^7_k \left( \mu \right) d \mu , \\ R \left( t_{n + 1} \right)= & {} R \left( 0 \right) + \frac{\tau }{\Gamma \left( \eta \right) } \sum _{k = 0}^{n} \int _{t_k}^{t_{k+1}} \mu ^{\tau - 1} \left( t_{n + 1} - \mu \right) ^{\eta - 1} P^8_k \left( \mu \right) d \mu , \end{aligned}$$

By resolving the integrals on the right-hand sides, the numerical scheme shown below is obtained.

$$\begin{aligned} S \left( t_{n + 1} \right)= & {} S \left( 0 \right) + \frac{\tau \left( \Delta t \right) ^\eta }{\Gamma \left( \eta + 2 \right) } \sum _{k = 0}^{n} \left[ t^{\tau - 1}_k F_1\left( t_k, S \left( t_k \right) \right) \times \left( \left( n + 1 -k \right) ^\eta \left( n - k + 2 + \eta \right) - \left( n - k \right) ^\eta \left( n - k + 2 + 2 \eta \right) \right) \right. \\{} & {} \left. - t^{\tau - 1}_{k-1} F_1\left( t_{k-1}, S \left( t_{k-1} \right) \right) \times \left( \left( n + 1 - k \right) ^{\eta + 1} - \left( n - k \right) ^\eta \left( n - k + 1 + \eta \right) \right) \right] , \\ E \left( t_{n + 1} \right)= & {} E \left( 0 \right) + \frac{\tau \left( \Delta t \right) ^\eta }{\Gamma \left( \eta + 2 \right) } \sum _{k = 0}^{n} \left[ t^{\tau - 1}_k F_2\left( t_k, E \left( t_k \right) \right) \times \left( \left( n + 1 -k \right) ^\eta \left( n - k + 2 + \eta \right) - \left( n - k \right) ^\eta \left( n - k + 2 + 2 \eta \right) \right) \right. \\ {}{} & {} \left. - t^{\tau - 1}_{k-1} F_2\left( t_{k-1}, E \left( t_{k-1} \right) \right) \times \left( \left( n + 1 - k \right) ^{\eta + 1} - \left( n - k \right) ^\eta \left( n - k + 1 + \eta \right) \right) \right] , \\ Q \left( t_{n + 1} \right)= & {} Q \left( 0 \right) + \frac{\tau \left( \Delta t \right) ^\eta }{\Gamma \left( \eta + 2 \right) } \sum _{k = 0}^{n} \left[ t^{\tau - 1}_k F_3\left( t_k, Q \left( t_k \right) \right) \times \left( \left( n + 1 -k \right) ^\eta \left( n - k + 2 + \eta \right) - \left( n - k \right) ^\eta \left( n - k + 2 + 2 \eta \right) \right) \right. \\ {}{} & {} \left. - t^{\tau - 1}_{k-1} F_3\left( t_{k-1}, Q \left( t_{k-1} \right) \right) \times \left( \left( n + 1 - k \right) ^{\eta + 1} - \left( n - k \right) ^\eta \left( n - k + 1 + \eta \right) \right) \right] , \\ I_A \left( t_{n + 1} \right)= & {} I_A \left( 0 \right) + \frac{\tau \left( \Delta t \right) ^\eta }{\Gamma \left( \eta + 2 \right) } \sum _{k = 0}^{n} \left[ t^{\tau - 1}_k F_4\left( t_k, I_A \left( t_k \right) \right) \times \left( \left( n + 1 -k \right) ^\eta \left( n - k + 2 + \eta \right) - \left( n - k \right) ^\eta \left( n - k + 2 + 2 \eta \right) \right) \right. \\ {}{} & {} \left. - t^{\tau - 1}_{k-1} F_4\left( t_{k-1}, I_A \left( t_{k-1} \right) \right) \times \left( \left( n + 1 - k \right) ^{\eta + 1} - \left( n - k \right) ^\eta \left( n - k + 1 + \eta \right) \right) \right] , \\ I_S \left( t_{n + 1} \right)= & {} I_S \left( 0 \right) + \frac{\tau \left( \Delta t \right) ^\eta }{\Gamma \left( \eta + 2 \right) } \sum _{k = 0}^{n} \left[ t^{\tau - 1}_k F_5\left( t_k, I_S \left( t_k \right) \right) \times \left( \left( n + 1 -k \right) ^\eta \left( n - k + 2 + \eta \right) - \left( n - k \right) ^\eta \left( n - k + 2 + 2 \eta \right) \right) \right. \\ {}{} & {} \left. - t^{\tau - 1}_{k-1} F_5\left( t_{k-1}, I_S \left( t_{k-1} \right) \right) \times \left( \left( n + 1 - k \right) ^{\eta + 1} - \left( n - k \right) ^\eta \left( n - k + 1 + \eta \right) \right) \right] , \\ H \left( t_{n + 1} \right)= & {} H \left( 0 \right) + \frac{\tau \left( \Delta t \right) ^\eta }{\Gamma \left( \eta + 2 \right) } \sum _{k = 0}^{n} \left[ t^{\tau - 1}_k F_6\left( t_k, H \left( t_k \right) \right) \times \left( \left( n + 1 -k \right) ^\eta \left( n - k + 2 + \eta \right) - \left( n - k \right) ^\eta \left( n - k + 2 + 2 \eta \right) \right) \right. \\ {}{} & {} \left. - t^{\tau - 1}_{k-1} F_6\left( t_{k-1}, H \left( t_{k-1} \right) \right) \times \left( \left( n + 1 - k \right) ^{\eta + 1} - \left( n - k \right) ^\eta \left( n - k + 1 + \eta \right) \right) \right] , \\ V \left( t_{n + 1} \right)= & {} V \left( 0 \right) + \frac{\tau \left( \Delta t \right) ^\eta }{\Gamma \left( \eta + 2 \right) } \sum _{k = 0}^{n} \left[ t^{\tau - 1}_k F_7\left( t_k, V \left( t_k \right) \right) \times \left( \left( n + 1 -k \right) ^\eta \left( n - k + 2 + \eta \right) - \left( n - k \right) ^\eta \left( n - k + 2 + 2 \eta \right) \right) \right. \\ {}{} & {} \left. - t^{\tau - 1}_{k-1} F_7\left( t_{k-1}, V \left( t_{k-1} \right) \right) \times \left( \left( n + 1 - k \right) ^{\eta + 1} - \left( n - k \right) ^\eta \left( n - k + 1 + \eta \right) \right) \right] , \\ R \left( t_{n + 1} \right)= & {} R \left( 0 \right) + \frac{\tau \left( \Delta t \right) ^\eta }{\Gamma \left( \eta + 2 \right) } \sum _{k = 0}^{n} \left[ t^{\tau - 1}_k F_8\left( t_k, R \left( t_k \right) \right) \times \left( \left( n + 1 -k \right) ^\eta \left( n - k + 2 + \eta \right) - \left( n - k \right) ^\eta \left( n - k + 2 + 2 \eta \right) \right) \right. \\ {}{} & {} \left. - t^{\tau - 1}_{k-1} F_8\left( t_{k-1}, R \left( t_{k-1} \right) \right) \times \left( \left( n + 1 - k \right) ^{\eta + 1} - \left( n - k \right) ^\eta \left( n - k + 1 + \eta \right) \right) \right] , \end{aligned}$$

**Figure 5:**
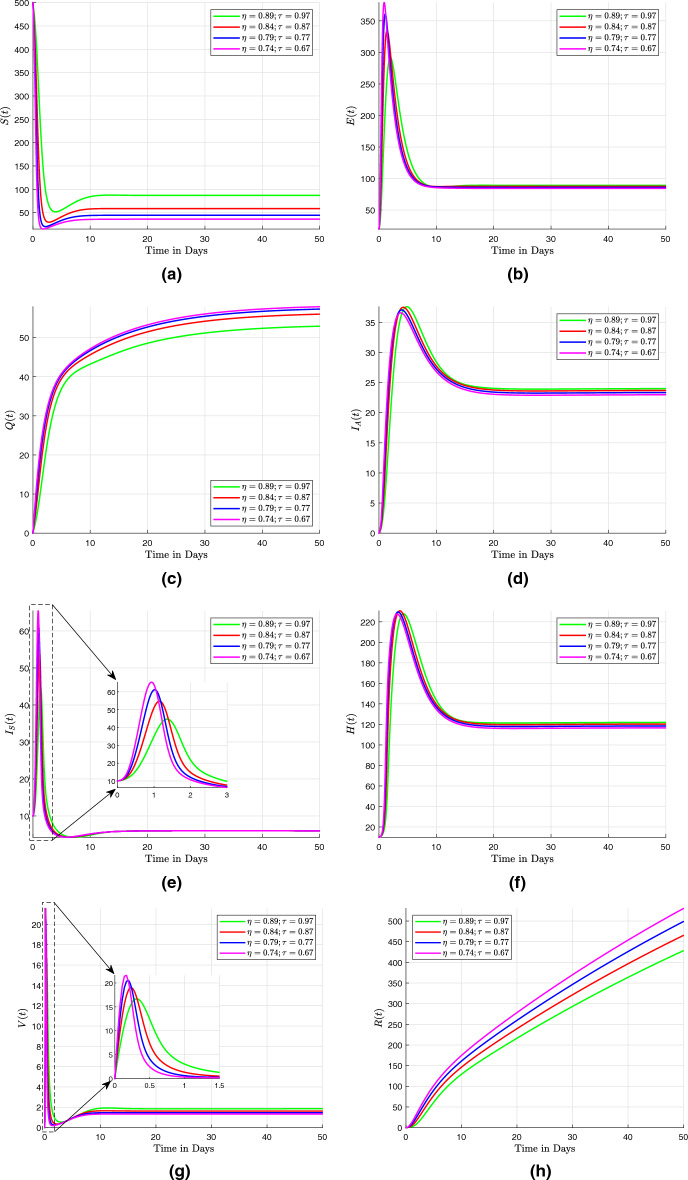
The dynamical behavior of the (**a**) susceptible population density; (**b**) exposed population density; (**c**) quarantined population density; (**d**) asymptomatically infected population density; (**e**) symptomatically infected population density; (**f**) hospitalized population density; (**g**) vaccinated population density; and (**h**) recovered population density; for fractal-fractional derivative in Caputo sense.

The progression of a disease model is illustrated in Fig. 5 through the dynamic behaviour of various population densities. Different variables indicate different stages or conditions in the population regarding the disease. In order to understand how these population densities change over time, the model employs fractal-fractional derivatives in the Caputo sense, which is a mathematical strategy that combines fractal and fractional calculus. This approach allows for a more accurate representation of the complex dynamics involved in disease progression, offering insights into potential interventions and control measures. By incorporating fractal-fractional derivatives, researchers can better predict and analyze the spread of diseases within populations.

### Fractal-fractional derivative in Caputo–Fabrizio sense

Here, we take into account the model (29) and the fractal fractional operators are defined in the Caputo Fabrizio sense. As a result, the first model, let us say, can be transformed into the following.

$$\begin{aligned} ^{FFCF}D^{\eta }_{0,t} \left\{ S \left( t \right) \right\}= & {} \tau t^{\tau - 1} F_1\left( t, S \left( t \right) \right) , \\ ^{FFCF}D^{\eta }_{0,t} \left\{ E \left( t \right) \right\}= & {} \tau t^{\tau - 1} F_2\left( t, E \left( t \right) \right) , \\ ^{FFCF}D^{\eta }_{0,t} \left\{ Q \left( t \right) \right\}= & {} \tau t^{\tau - 1} F_3\left( t, Q \left( t \right) \right) , \\ ^{FFCF}D^{\eta }_{0,t} \left\{ I_A \left( t \right) \right\}= & {} \tau t^{\tau - 1} F_4\left( t, I_A \left( t \right) \right) , \\ ^{FFCF}D^{\eta }_{0,t} \left\{ I_S \left( t \right) \right\}= & {} \tau t^{\tau - 1} F_5\left( t, I_S \left( t \right) \right) , \\ ^{FFCF}D^{\eta }_{0,t} \left\{ H \left( t \right) \right\}= & {} \tau t^{\tau - 1} F_6\left( t, H \left( t \right) \right) , \\ ^{FFCF}D^{\eta }_{0,t} \left\{ V \left( t \right) \right\}= & {} \tau t^{\tau - 1} F_7\left( t, V \left( t \right) \right) , \\ ^{FFCF}D^{\eta }_{0,t} \left\{ R \left( t \right) \right\}= & {} \tau t^{\tau - 1} F_8\left( t, R \left( t \right) \right) , \end{aligned}$$

By utilizing the Caputo–Fabrizio integral, we arrive at

$$\begin{aligned} S \left( t \right)= & {} S \left( 0 \right) + \frac{\tau t^{\tau - 1} \left( 1 - \eta \right) }{ M\left( \eta \right) } F_1 \left( t, S \left( t \right) \right) + \frac{\eta \tau }{M \left( \eta \right) } \int _{0}^{t} \mu ^{\tau - 1} F_1 \left( \mu , S \left( \mu \right) \right) d \mu , \\ E \left( t \right)= & {} E \left( 0 \right) + \frac{\tau t^{\tau - 1} \left( 1 - \eta \right) }{ M\left( \eta \right) } F_2 \left( t, E \left( t \right) \right) + \frac{\eta \tau }{M \left( \eta \right) } \int _{0}^{t} \mu ^{\tau - 1} F_2 \left( \mu , E \left( \mu \right) \right) d \mu , \\ Q \left( t \right)= & {} Q \left( 0 \right) + \frac{\tau t^{\tau - 1} \left( 1 - \eta \right) }{ M\left( \eta \right) } F_3 \left( t, Q \left( t \right) \right) + \frac{\eta \tau }{M \left( \eta \right) } \int _{0}^{t} \mu ^{\tau - 1} F_3 \left( \mu , Q \left( \mu \right) \right) d \mu , \\ I_A \left( t \right)= & {} I_A \left( 0 \right) + \frac{\tau t^{\tau - 1} \left( 1 - \eta \right) }{ M\left( \eta \right) } F_4 \left( t, I_A \left( t \right) \right) + \frac{\eta \tau }{M \left( \eta \right) } \int _{0}^{t} \mu ^{\tau - 1} F_4 \left( \mu , I_A \left( \mu \right) \right) d \mu , \\ I_S \left( t \right)= & {} I_S \left( 0 \right) + \frac{\tau t^{\tau - 1} \left( 1 - \eta \right) }{ M\left( \eta \right) } F_5 \left( t, I_S \left( t \right) \right) + \frac{\eta \tau }{M \left( \eta \right) } \int _{0}^{t} \mu ^{\tau - 1} F_5 \left( \mu , I_S \left( \mu \right) \right) d \mu , \\ H \left( t \right)= & {} H \left( 0 \right) + \frac{\tau t^{\tau - 1} \left( 1 - \eta \right) }{ M\left( \eta \right) } F_6 \left( t, H \left( t \right) \right) + \frac{\eta \tau }{M \left( \eta \right) } \int _{0}^{t} \mu ^{\tau - 1} F_6 \left( \mu , H \left( \mu \right) \right) d \mu , \\ V \left( t \right)= & {} V \left( 0 \right) + \frac{\tau t^{\tau - 1} \left( 1 - \eta \right) }{ M\left( \eta \right) } F_7 \left( t, V \left( t \right) \right) + \frac{\eta \tau }{M \left( \eta \right) } \int _{0}^{t} \mu ^{\tau - 1} F_7 \left( \mu , V \left( \mu \right) \right) d \mu , \\ R \left( t \right)= & {} R \left( 0 \right) + \frac{\tau t^{\tau - 1} \left( 1 - \eta \right) }{ M\left( \eta \right) } F_8 \left( t, R \left( t \right) \right) + \frac{\eta \tau }{M \left( \eta \right) } \int _{0}^{t} \mu ^{\tau - 1} F_8 \left( \mu , R \left( \mu \right) \right) d \mu , \end{aligned}$$

Here, we outline the numerical scheme’s thorough derivation. Thus, at $t_{n + 1}$ we have

$$\begin{aligned} S \left( t_{n+1} \right)= & {} S \left( t_0 \right) + \frac{\tau t^{\tau - 1}_n \left( 1 - \eta \right) }{ M\left( \eta \right) } F_1 \left( t_n, S \left( t_n \right) \right) + \frac{\eta \tau }{M \left( \eta \right) } \int _{t_0}^{t_{n+1}} \mu ^{\tau - 1} F_1 \left( \mu , S \left( \mu \right) \right) d \mu , \\ E \left( t_{n+1} \right)= & {} E \left( t_0 \right) + \frac{\tau t^{\tau - 1}_n \left( 1 - \eta \right) }{ M\left( \eta \right) } F_2 \left( t_n, E \left( t_n \right) \right) + \frac{\eta \tau }{M \left( \eta \right) } \int _{t_0}^{t_{n+1}} \mu ^{\tau - 1} F_2 \left( \mu , E \left( \mu \right) \right) d \mu , \\ Q \left( t_{n+1} \right)= & {} Q \left( t_0 \right) + \frac{\tau t^{\tau - 1}_n \left( 1 - \eta \right) }{ M\left( \eta \right) } F_3 \left( t_n, Q \left( t_n \right) \right) + \frac{\eta \tau }{M \left( \eta \right) } \int _{t_0}^{t_{n+1}} \mu ^{\tau - 1} F_3 \left( \mu , Q \left( \mu \right) \right) d \mu , \\ I_A \left( t_{n+1} \right)= & {} I_A \left( t_0 \right) + \frac{\tau t^{\tau - 1}_n \left( 1 - \eta \right) }{ M\left( \eta \right) } F_4 \left( t_n, I_A \left( t_n \right) \right) + \frac{\eta \tau }{M \left( \eta \right) } \int _{t_0}^{t_{n+1}} \mu ^{\tau - 1} F_4 \left( \mu , I_A \left( \mu \right) \right) d \mu , \\ I_S \left( t_{n+1} \right)= & {} I_S \left( t_0 \right) + \frac{\tau t^{\tau - 1}_n \left( 1 - \eta \right) }{ M\left( \eta \right) } F_5 \left( t_n, I_S \left( t_n \right) \right) + \frac{\eta \tau }{M \left( \eta \right) } \int _{t_0}^{t_{n+1}} \mu ^{\tau - 1} F_5 \left( \mu , I_S \left( \mu \right) \right) d \mu , \\ H \left( t_{n+1} \right)= & {} H \left( t_0 \right) + \frac{\tau t^{\tau - 1}_n \left( 1 - \eta \right) }{ M\left( \eta \right) } F_6 \left( t_n, H \left( t_n \right) \right) + \frac{\eta \tau }{M \left( \eta \right) } \int _{t_0}^{t_{n+1}} \mu ^{\tau - 1} F_6 \left( \mu , H \left( \mu \right) \right) d \mu , \\ V \left( t_{n+1} \right)= & {} V \left( t_0 \right) + \frac{\tau t^{\tau - 1}_n \left( 1 - \eta \right) }{ M\left( \eta \right) } F_7 \left( t_n, V \left( t_n \right) \right) + \frac{\eta \tau }{M \left( \eta \right) } \int _{t_0}^{t_{n+1}} \mu ^{\tau - 1} F_7 \left( \mu , V \left( \mu \right) \right) d \mu , \\ R \left( t_{n+1} \right)= & {} R \left( t_0 \right) + \frac{\tau t^{\tau - 1}_n \left( 1 - \eta \right) }{ M\left( \eta \right) } F_8 \left( t_n, R \left( t_n \right) \right) + \frac{\eta \tau }{M \left( \eta \right) } \int _{t_0}^{t_{n+1}} \mu ^{\tau - 1} F_8 \left( \mu , R \left( \mu \right) \right) d \mu , \end{aligned}$$

Finding the gap between two consecutive terms yields

$$\begin{aligned} S \left( t_{n+1} \right)= & {} S \left( t_n \right) + \frac{\tau t^{\tau - 1}_n \left( 1 - \eta \right) }{ M\left( \eta \right) } F_1 \left( t_n, S \left( t_n \right) \right) - \frac{\tau t^{\tau - 1}_{n-1} \left( 1 - \eta \right) }{ M\left( \eta \right) } F_1 \left( t_{n-1}, S \left( t_{n-1} \right) \right) + \frac{\eta \tau }{M \left( \eta \right) } \int _{t_n}^{t_{n+1}} \mu ^{\tau - 1} F_1 \left( \mu , S \left( \mu \right) \right) d \mu , \\ E \left( t_{n+1} \right)= & {} E \left( t_n \right) + \frac{\tau t^{\tau - 1}_n \left( 1 - \eta \right) }{ M\left( \eta \right) } F_2 \left( t_n, E \left( t_n \right) \right) - \frac{\tau t^{\tau - 1}_{n-1} \left( 1 - \eta \right) }{ M\left( \eta \right) } F_2 \left( t_{n-1}, E \left( t_{n-1} \right) \right) + \frac{\eta \tau }{M \left( \eta \right) } \int _{t_n}^{t_{n+1}} \mu ^{\tau - 1} F_2 \left( \mu , E \left( \mu \right) \right) d \mu , \\ Q \left( t_{n+1} \right)= & {} Q \left( t_n \right) + \frac{\tau t^{\tau - 1}_n \left( 1 - \eta \right) }{ M\left( \eta \right) } F_3 \left( t_n, Q \left( t_n \right) \right) - \frac{\tau t^{\tau - 1}_{n-1} \left( 1 - \eta \right) }{ M\left( \eta \right) } F_3 \left( t_{n-1}, Q \left( t_{n-1} \right) \right) + \frac{\eta \tau }{M \left( \eta \right) } \int _{t_n}^{t_{n+1}} \mu ^{\tau - 1} F_3 \left( \mu , Q \left( \mu \right) \right) d \mu , \\ I_A \left( t_{n+1} \right)= & {} I_A \left( t_n \right) + \frac{\tau t^{\tau - 1}_n \left( 1 - \eta \right) }{ M\left( \eta \right) } F_4 \left( t_n, I_A \left( t_n \right) \right) - \frac{\tau t^{\tau - 1}_{n-1} \left( 1 - \eta \right) }{ M\left( \eta \right) } F_4 \left( t_{n-1}, I_A \left( t_{n-1} \right) \right) + \frac{\eta \tau }{M \left( \eta \right) } \int _{t_n}^{t_{n+1}} \mu ^{\tau - 1} F_4 \left( \mu , I_A \left( \mu \right) \right) d \mu , \\ I_S \left( t_{n+1} \right)= & {} I_S \left( t_n \right) + \frac{\tau t^{\tau - 1}_n \left( 1 - \eta \right) }{ M\left( \eta \right) } F_5 \left( t_n, I_S \left( t_n \right) \right) - \frac{\tau t^{\tau - 1}_{n-1} \left( 1 - \eta \right) }{ M\left( \eta \right) } F_5 \left( t_{n-1}, I_S \left( t_{n-1} \right) \right) + \frac{\eta \tau }{M \left( \eta \right) } \int _{t_n}^{t_{n+1}} \mu ^{\tau - 1} F_5 \left( \mu , I_S \left( \mu \right) \right) d \mu , \\ H \left( t_{n+1} \right)= & {} H \left( t_n \right) + \frac{\tau t^{\tau - 1}_n \left( 1 - \eta \right) }{ M\left( \eta \right) } F_6 \left( t_n, H \left( t_n \right) \right) - \frac{\tau t^{\tau - 1}_{n-1} \left( 1 - \eta \right) }{ M\left( \eta \right) } F_6 \left( t_{n-1}, H \left( t_{n-1} \right) \right) + \frac{\eta \tau }{M \left( \eta \right) } \int _{t_n}^{t_{n+1}} \mu ^{\tau - 1} F_6 \left( \mu , H \left( \mu \right) \right) d \mu , \\ V \left( t_{n+1} \right)= & {} V \left( t_n \right) + \frac{\tau t^{\tau - 1}_n \left( 1 - \eta \right) }{ M\left( \eta \right) } F_7 \left( t_n, V \left( t_n \right) \right) - \frac{\tau t^{\tau - 1}_{n-1} \left( 1 - \eta \right) }{ M\left( \eta \right) } F_7 \left( t_{n-1}, V \left( t_{n-1} \right) \right) + \frac{\eta \tau }{M \left( \eta \right) } \int _{t_n}^{t_{n+1}} \mu ^{\tau - 1} F_7 \left( \mu , V \left( \mu \right) \right) d \mu , \\ R \left( t_{n+1} \right)= & {} R \left( t_n \right) + \frac{\tau t^{\tau - 1}_n \left( 1 - \eta \right) }{ M\left( \eta 
\right) } F_8 \left( t_n, R \left( t_n \right) \right) - \frac{\tau t^{\tau - 1}_{n-1} \left( 1 - \eta \right) }{ M\left( \eta \right) } F_8 \left( t_{n-1}, R \left( t_{n-1} \right) \right) + \frac{\eta \tau }{M \left( \eta \right) } \int _{t_n}^{t_{n+1}} \mu ^{\tau - 1} F_8 \left( \mu , R \left( \mu \right) \right) d \mu , \end{aligned}$$

Finally, we integrate and perform piecewise interpolation using the Lagrange polynomial to get

$$\begin{aligned} S \left( t_{n+1} \right)= & {} S \left( t_n \right) + \frac{\tau t^{\tau - 1}_n \left( 1 - \eta \right) }{ M\left( \eta \right) } F_1 \left( t_n, S \left( t_n \right) \right) - \frac{\tau t^{\tau - 1}_{n-1} \left( 1 - \eta \right) }{ M\left( \eta \right) } F_1 \left( t_{n-1}, S \left( t_{n-1} \right) \right) \\{} & {} + \frac{\eta \tau }{M \left( \eta \right) } \times \left[ \frac{3}{2} \left( \Delta t \right) t^{\tau - 1}_n F_1 \left( t_n, S \left( t_n \right) \right) - \frac{\Delta t}{2} t^{\tau - 1}_{n - 1} F_1 \left( t_{n-1}, S \left( t_{n-1} \right) \right) \right] , \\ E \left( t_{n+1} \right)= & {} E \left( t_n \right) + \frac{\tau t^{\tau - 1}_n \left( 1 - \eta \right) }{ M\left( \eta \right) } F_2 \left( t_n, E \left( t_n \right) \right) - \frac{\tau t^{\tau - 1}_{n-1} \left( 1 - \eta \right) }{ M\left( \eta \right) } F_2 \left( t_{n-1}, E \left( t_{n-1} \right) \right) \\{} & {} +\frac{\eta \tau }{M \left( \eta \right) } \times \left[ \frac{3}{2} \left( \Delta t \right) t^{\tau - 1}_n F_2 \left( t_n, E \left( t_n \right) \right) - \frac{\Delta t}{2} t^{\tau - 1}_{n - 1} F_2 \left( t_{n-1}, E \left( t_{n-1} \right) \right) \right] , \\ Q \left( t_{n+1} \right)= & {} Q \left( t_n \right) + \frac{\tau t^{\tau - 1}_n \left( 1 - \eta \right) }{ M\left( \eta \right) } F_3 \left( t_n, Q \left( t_n \right) \right) - \frac{\tau t^{\tau - 1}_{n-1} \left( 1 - \eta \right) }{ M\left( \eta \right) } F_3 \left( t_{n-1}, Q \left( t_{n-1} \right) \right) \\{} & {} +\frac{\eta \tau }{M \left( \eta \right) } \times \left[ \frac{3}{2} \left( \Delta t \right) t^{\tau - 1}_n F_3 \left( t_n, Q \left( t_n \right) \right) - \frac{\Delta t}{2} t^{\tau - 1}_{n - 1} F_3 \left( t_{n-1}, Q \left( t_{n-1} \right) \right) \right] , \\ I_A \left( t_{n+1} \right)= & {} I_A \left( t_n \right) + \frac{\tau t^{\tau - 1}_n \left( 1 - \eta \right) }{ M\left( \eta \right) } F_4 \left( t_n, I_A \left( t_n \right) \right) - \frac{\tau t^{\tau - 1}_{n-1} \left( 1 - \eta \right) }{ M\left( \eta \right) } F_4 \left( t_{n-1}, I_A \left( t_{n-1} \right) \right) \\{} & {} +\frac{\eta \tau }{M \left( \eta \right) } \times \left[ \frac{3}{2} \left( \Delta t \right) t^{\tau - 1}_n F_4 \left( t_n, I_A \left( t_n \right) \right) - \frac{\Delta t}{2} t^{\tau - 1}_{n - 1} F_4 \left( t_{n-1}, I_A \left( t_{n-1} \right) \right) \right] , \\ I_S \left( t_{n+1} \right)= & {} I_S \left( t_n \right) + \frac{\tau t^{\tau - 1}_n \left( 1 - \eta \right) }{ M\left( \eta \right) } F_5 \left( t_n, I_S \left( t_n \right) \right) - \frac{\tau t^{\tau - 1}_{n-1} \left( 1 - \eta \right) }{ M\left( \eta \right) } F_5 \left( t_{n-1}, I_S \left( t_{n-1} \right) \right) \\{} & {} +\frac{\eta \tau }{M \left( \eta \right) } \times \left[ \frac{3}{2} \left( \Delta t \right) t^{\tau - 1}_n F_5 \left( t_n, I_S \left( t_n \right) \right) - \frac{\Delta t}{2} t^{\tau - 1}_{n - 1} F_5 \left( t_{n-1}, I_S \left( t_{n-1} \right) \right) \right] , \\ H \left( t_{n+1} \right)= & {} H \left( t_n \right) + \frac{\tau t^{\tau - 1}_n \left( 1 - \eta \right) }{ M\left( \eta \right) } F_6 \left( t_n, H \left( t_n \right) \right) - \frac{\tau t^{\tau - 1}_{n-1} \left( 1 - \eta \right) }{ M\left( \eta \right) } F_6 \left( t_{n-1}, H \left( t_{n-1} \right) \right) \\{} & {} +\frac{\eta \tau }{M \left( \eta \right) } \times \left[ \frac{3}{2} \left( \Delta t \right) t^{\tau - 1}_n F_6 \left( t_n, H \left( t_n \right) \right) - \frac{\Delta t}{2} t^{\tau - 1}_{n - 1} F_6 \left( t_{n-1}, H \left( t_{n-1} \right) \right) \right] , \\ V \left( t_{n+1} \right)= & {} V \left( t_n \right) + \frac{\tau t^{\tau - 1}_n \left( 1 - \eta \right) }{ M\left( \eta \right) } F_7 \left( t_n, V \left( t_n \right) \right) - \frac{\tau t^{\tau - 1}_{n-1} \left( 1 - \eta \right) }{ M\left( \eta \right) } F_7 \left( t_{n-1}, V \left( t_{n-1} \right) \right) \\{} & {} +\frac{\eta \tau }{M \left( \eta \right) } \times \left[ \frac{3}{2} \left( \Delta t \right) t^{\tau - 1}_n F_7 \left( t_n, V \left( t_n \right) \right) - \frac{\Delta t}{2} t^{\tau - 1}_{n - 1} F_7 \left( t_{n-1}, V \left( t_{n-1} \right) \right) \right] , \\ R \left( t_{n+1} \right)= & {} R \left( t_n \right) + \frac{\tau t^{\tau - 1}_n \left( 1 - \eta \right) }{ M\left( \eta \right) } F_8 \left( t_n, R \left( t_n \right) \right) - \frac{\tau t^{\tau - 1}_{n-1} \left( 1 - \eta \right) }{ M\left( \eta \right) } F_8 \left( t_{n-1}, R \left( t_{n-1} \right) \right) \\{} & {} + \frac{\eta \tau }{M \left( \eta \right) } \times \left[ \frac{3}{2} \left( \Delta t \right) t^{\tau - 1}_n F_8 \left( t_n, R \left( t_n \right) \right) - \frac{\Delta t}{2} t^{\tau - 1}_{n - 1} F_8 \left( t_{n-1}, R \left( t_{n-1} \right) \right) \right] , \end{aligned}$$

**Figure 6:**
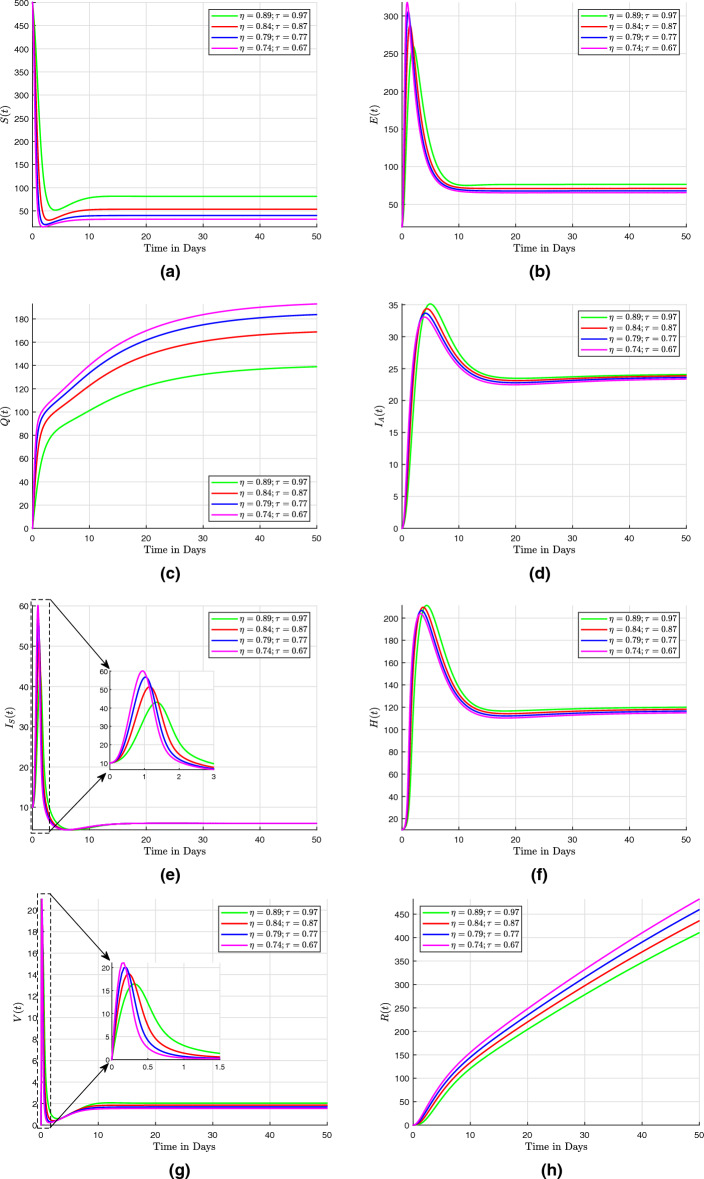
The dynamical behavior of the (**a**) susceptible population density; (**b**) exposed population density; (**c**) quarantined population density; (**d**) asymptomatically infected population density; (**e**) symptomatically infected population density; (**f**) hospitalized population density; (**g**) vaccinated population density; and (h) recovered population density; for fractal-fractional derivative in Caputo–Fabrizio sense.

In order to understand the temporal changes in these population densities, the model employs fractal-fractional derivatives in the Caputo–Fabrizio sense, a mathematical technique that combines fractal and fractional calculus. This is achieved by analysing the changing behaviour of different population densities, as demonstrated in Fig. 6. This method converges to a more accurate representation of the complex dynamics of population changes over time, allowing for more precise predictions and analysis. Additionally, it provides a unique perspective on the interplay between fractal geometry and fractional calculus in ecological modeling. This method makes it easier to show the complex dynamics of how diseases spread, which can help with coming up with new ways to treat and prevent them. Furthermore, by incorporating fractal geometry and fractional calculus, this approach enhances our understanding of the underlying mechanisms driving population dynamics. It also opens up new avenues for exploring the intricate relationships between ecological systems and mathematical modeling. Incorporating fractal-fractional derivatives helps researchers predict and study how diseases spread within populations more accurately.

### Fractal-fractional derivative in Atangana–Baleanu sense

In this section, we take into account the model (29) with the fractal-fractional differential operator in Atangana–Baleanu sense. Using the model (29) in this context, we can write

$$\begin{aligned} ^{FFABC}D^{\eta }_{0,t} \left\{ S \left( t \right) \right\}= & {} \tau t^{\tau - 1} F_1\left( t, S \left( t \right) \right) , \\ ^{FFABC}D^{\eta }_{0,t} \left\{ E \left( t \right) \right\}= & {} \tau t^{\tau - 1} F_2\left( t, E \left( t \right) \right) , \\ ^{FFABC}D^{\eta }_{0,t} \left\{ Q \left( t \right) \right\}= & {} \tau t^{\tau - 1} F_3\left( t, Q \left( t \right) \right) , \\ ^{FFABC}D^{\eta }_{0,t} \left\{ I_A \left( t \right) \right\}= & {} \tau t^{\tau - 1} F_4\left( t, I_A \left( t \right) \right) , \\ ^{FFABC}D^{\eta }_{0,t} \left\{ I_S \left( t \right) \right\}= & {} \tau t^{\tau - 1} F_5\left( t, I_S \left( t \right) \right) , \\ ^{FFABC}D^{\eta }_{0,t} \left\{ H \left( t \right) \right\}= & {} \tau t^{\tau - 1} F_6\left( t, H \left( t \right) \right) , \\ ^{FFABC}D^{\eta }_{0,t} \left\{ V \left( t \right) \right\}= & {} \tau t^{\tau - 1} F_7\left( t, V \left( t \right) \right) , \\ ^{FFABC}D^{\eta }_{0,t} \left\{ R \left( t \right) \right\}= & {} \tau t^{\tau - 1} F_8\left( t, R \left( t \right) \right) , \end{aligned}$$

This is extended to the Atangana–Baleanu in the Caputo sense, and the Atangana–Baleanu integral is used to arrive at the following:

$$\begin{aligned} S \left( t \right)= & {} S \left( 0 \right) + \frac{\tau t^{\tau - 1} \left( 1 - \eta \right) }{ AB\left( \eta \right) } F_1 \left( t, S \left( t \right) \right) + \frac{\eta \tau }{AB \left( \eta \right) \Gamma \left( \eta \right) } \int _{0}^{t} \mu ^{\tau - 1} \left( t - \mu \right) ^{\eta - 1} F_1 \left( \mu , S \left( \mu \right) \right) d \mu , \\ E \left( t \right)= & {} E \left( 0 \right) + \frac{\tau t^{\tau - 1} \left( 1 - \eta \right) }{ AB\left( \eta \right) } F_2 \left( t, E \left( t \right) \right) + \frac{\eta \tau }{AB \left( \eta \right) \Gamma \left( \eta \right) } \int _{0}^{t} \mu ^{\tau - 1} \left( t - \mu \right) ^{\eta - 1} F_2 \left( \mu , E \left( \mu \right) \right) d \mu , \\ Q \left( t \right)= & {} Q \left( 0 \right) + \frac{\tau t^{\tau - 1} \left( 1 - \eta \right) }{ AB\left( \eta \right) } F_3 \left( t, Q \left( t \right) \right) + \frac{\eta \tau }{AB \left( \eta \right) \Gamma \left( \eta \right) } \int _{0}^{t} \mu ^{\tau - 1} \left( t - \mu \right) ^{\eta - 1} F_3 \left( \mu , Q \left( \mu \right) \right) d \mu , \\ I_A \left( t \right)= & {} I_A \left( 0 \right) + \frac{\tau t^{\tau - 1} \left( 1 - \eta \right) }{ AB\left( \eta \right) } F_4 \left( t, I_A \left( t \right) \right) + \frac{\eta \tau }{AB \left( \eta \right) \Gamma \left( \eta \right) } \int _{0}^{t} \mu ^{\tau - 1} \left( t - \mu \right) ^{\eta - 1} F_4 \left( \mu , I_A \left( \mu \right) \right) d \mu , \\ I_S \left( t \right)= & {} I_S \left( 0 \right) + \frac{\tau t^{\tau - 1} \left( 1 - \eta \right) }{ AB\left( \eta \right) } F_5 \left( t, I_S \left( t \right) \right) + \frac{\eta \tau }{AB \left( \eta \right) \Gamma \left( \eta \right) } \int _{0}^{t} \mu ^{\tau - 1} \left( t - \mu \right) ^{\eta - 1} F_5 \left( \mu , I_S \left( \mu \right) \right) d \mu , \\ H \left( t \right)= & {} H \left( 0 \right) + \frac{\tau t^{\tau - 1} \left( 1 - \eta \right) }{ AB\left( \eta \right) } F_6 \left( t, H \left( t \right) \right) + \frac{\eta \tau }{AB \left( \eta \right) \Gamma \left( \eta \right) } \int _{0}^{t} \mu ^{\tau - 1} \left( t - \mu \right) ^{\eta - 1} F_6 \left( \mu , H \left( \mu \right) \right) d \mu , \\ V \left( t \right)= & {} V \left( 0 \right) + \frac{\tau t^{\tau - 1} \left( 1 - \eta \right) }{ AB\left( \eta \right) } F_7 \left( t, V \left( t \right) \right) + \frac{\eta \tau }{AB \left( \eta \right) \Gamma \left( \eta \right) } \int _{0}^{t} \mu ^{\tau - 1} \left( t - \mu \right) ^{\eta - 1} F_7 \left( \mu , V \left( \mu \right) \right) d \mu , \\ R \left( t \right)= & {} R \left( 0 \right) + \frac{\tau t^{\tau - 1} \left( 1 - \eta \right) }{ AB\left( \eta \right) } F_8 \left( t, R \left( t \right) \right) + \frac{\eta \tau }{AB \left( \eta \right) \Gamma \left( \eta \right) } \int _{0}^{t} \mu ^{\tau - 1} \left( t - \mu \right) ^{\eta - 1} F_8 \left( \mu , R \left( \mu \right) \right) d \mu , \end{aligned}$$

At $t_{n + 1}$ , we have the following

$$\begin{aligned} S \left( t_{n+1} \right)= & {} S \left( t_0 \right) + \frac{\tau t^{\tau - 1}_n \left( 1 - \eta \right) }{ AB\left( \eta \right) } F_1 \left( t_n, S \left( t_n \right) \right) + \frac{\eta \tau }{AB \left( \eta \right) \Gamma \left( \eta \right) } \int _{t_0}^{t_{n+1}} \mu ^{\tau - 1} \left( t_{n+1} - \mu \right) ^{\eta - 1} F_1 \left( \mu , S \left( \mu \right) \right) d \mu , \\ E \left( t_{n+1} \right)= & {} E \left( t_0 \right) + \frac{\tau t^{\tau - 1}_n \left( 1 - \eta \right) }{ AB\left( \eta \right) } F_2 \left( t_n, E \left( t_n \right) \right) + \frac{\eta \tau }{AB \left( \eta \right) \Gamma \left( \eta \right) } \int _{t_0}^{t_{n+1}} \mu ^{\tau - 1} \left( t_{n+1} - \mu \right) ^{\eta - 1} F_2 \left( \mu , E \left( \mu \right) \right) d \mu , \\ Q \left( t_{n+1} \right)= & {} Q \left( t_0 \right) + \frac{\tau t^{\tau - 1}_n \left( 1 - \eta \right) }{ AB\left( \eta \right) } F_3 \left( t_n, Q \left( t_n \right) \right) + \frac{\eta \tau }{AB \left( \eta \right) \Gamma \left( \eta \right) } \int _{t_0}^{t_{n+1}} \mu ^{\tau - 1} \left( t_{n+1} - \mu \right) ^{\eta - 1} F_3 \left( \mu , Q \left( \mu \right) \right) d \mu , \\ I_A \left( t_{n+1} \right)= & {} I_A \left( t_0 \right) + \frac{\tau t^{\tau - 1}_n \left( 1 - \eta \right) }{ AB\left( \eta \right) } F_4 \left( t_n, I_A \left( t_n \right) \right) + \frac{\eta \tau }{AB \left( \eta \right) \Gamma \left( \eta \right) } \int _{t_0}^{t_{n+1}} \mu ^{\tau - 1} \left( t_{n+1} - \mu \right) ^{\eta - 1} F_4 \left( \mu , I_A \left( \mu \right) \right) d \mu , \\ I_S \left( t_{n+1} \right)= & {} I_S \left( t_0 \right) + \frac{\tau t^{\tau - 1}_n \left( 1 - \eta \right) }{ AB\left( \eta \right) } F_5 \left( t_n, I_S \left( t_n \right) \right) + \frac{\eta \tau }{AB \left( \eta \right) \Gamma \left( \eta \right) } \int _{t_0}^{t_{n+1}} \mu ^{\tau - 1} \left( t_{n+1} - \mu \right) ^{\eta - 1} F_5 \left( \mu , I_S \left( \mu \right) \right) d \mu , \\ H \left( t_{n+1} \right)= & {} H \left( t_0 \right) + \frac{\tau t^{\tau - 1}_n \left( 1 - \eta \right) }{ AB\left( \eta \right) } F_6 \left( t_n, H \left( t_n \right) \right) + \frac{\eta \tau }{AB \left( \eta \right) \Gamma \left( \eta \right) } \int _{t_0}^{t_{n+1}} \mu ^{\tau - 1} \left( t_{n+1} - \mu \right) ^{\eta - 1} F_6 \left( \mu , H \left( \mu \right) \right) d \mu , \\ V \left( t_{n+1} \right)= & {} V \left( t_0 \right) + \frac{\tau t^{\tau - 1}_n \left( 1 - \eta \right) }{ AB\left( \eta \right) } F_7 \left( t_n, V \left( t_n \right) \right) + \frac{\eta \tau }{AB \left( \eta \right) \Gamma \left( \eta \right) } \int _{t_0}^{t_{n+1}} \mu ^{\tau - 1} \left( t_{n+1} - \mu \right) ^{\eta - 1} F_7 \left( \mu , V \left( \mu \right) \right) d \mu , \\ R \left( t_{n+1} \right)= & {} R \left( t_0 \right) + \frac{\tau t^{\tau - 1}_n \left( 1 - \eta \right) }{ AB\left( \eta \right) } F_8 \left( t_n, R \left( t_n \right) \right) + \frac{\eta \tau }{AB \left( \eta \right) \Gamma \left( \eta \right) } \int _{t_0}^{t_{n+1}} \mu ^{\tau - 1} \left( t_{n+1} - \mu \right) ^{\eta - 1} F_8 \left( \mu , R \left( \mu \right) \right) d \mu , \end{aligned}$$

Approximating the integrals allows us to write down the above system as

$$\begin{aligned} S \left( t_{n+1} \right)= & {} S \left( t_0 \right) + \frac{\tau t^{\tau - 1}_n \left( 1 - \eta \right) }{ AB\left( \eta \right) } F_1 \left( t_n, S \left( t_n \right) \right) + \frac{\eta \tau }{AB \left( \eta \right) \Gamma \left( \eta \right) } \sum _{k=0}^{n} \int _{t_k}^{t_{k+1}} \mu ^{\tau - 1} \left( t_{n+1} - \mu \right) ^{\eta - 1} F_1 \left( \mu , S \left( \mu \right) \right) d \mu , \\ E \left( t_{n+1} \right)= & {} E \left( t_0 \right) + \frac{\tau t^{\tau - 1}_n \left( 1 - \eta \right) }{ AB\left( \eta \right) } F_2 \left( t_n, E \left( t_n \right) \right) + \frac{\eta \tau }{AB \left( \eta \right) \Gamma \left( \eta \right) } \sum _{k=0}^{n} \int _{t_k}^{t_{k+1}} \mu ^{\tau - 1} \left( t_{n+1} - \mu \right) ^{\eta - 1} F_2 \left( \mu , E \left( \mu \right) \right) d \mu , \\ Q \left( t_{n+1} \right)= & {} Q \left( t_0 \right) + \frac{\tau t^{\tau - 1}_n \left( 1 - \eta \right) }{ AB\left( \eta \right) } F_3 \left( t_n, Q \left( t_n \right) \right) + \frac{\eta \tau }{AB \left( \eta \right) \Gamma \left( \eta \right) } \sum _{k=0}^{n} \int _{t_k}^{t_{k+1}} \mu ^{\tau - 1} \left( t_{n+1} - \mu \right) ^{\eta - 1} F_3 \left( \mu , Q \left( \mu \right) \right) d \mu , \\ I_A \left( t_{n+1} \right)= & {} I_A \left( t_0 \right) + \frac{\tau t^{\tau - 1}_n \left( 1 - \eta \right) }{ AB\left( \eta \right) } F_4 \left( t_n, I_A \left( t_n \right) \right) + \frac{\eta \tau }{AB \left( \eta \right) \Gamma \left( \eta \right) } \sum _{k=0}^{n} \int _{t_k}^{t_{k+1}} \mu ^{\tau - 1} \left( t_{n+1} - \mu \right) ^{\eta - 1} F_4 \left( \mu , I_A \left( \mu \right) \right) d \mu , \\ I_S \left( t_{n+1} \right)= & {} I_S \left( t_0 \right) + \frac{\tau t^{\tau - 1}_n \left( 1 - \eta \right) }{ AB\left( \eta \right) } F_5 \left( t_n, I_S \left( t_n \right) \right) + \frac{\eta \tau }{AB \left( \eta \right) \Gamma \left( \eta \right) } \sum _{k=0}^{n} \int _{t_k}^{t_{k+1}} \mu ^{\tau - 1} \left( t_{n+1} - \mu \right) ^{\eta - 1} F_5 \left( \mu , I_S \left( \mu \right) \right) d \mu , \\ H \left( t_{n+1} \right)= & {} H \left( t_0 \right) + \frac{\tau t^{\tau - 1}_n \left( 1 - \eta \right) }{ AB\left( \eta 
\right) } F_6 \left( t_n, H \left( t_n \right) \right) + \frac{\eta \tau }{AB \left( \eta \right) \Gamma \left( \eta \right) } \sum _{k=0}^{n} \int _{t_k}^{t_{k+1}} \mu ^{\tau - 1} \left( t_{n+1} - \mu \right) ^{\eta - 1} F_6 \left( \mu , H \left( \mu \right) \right) d \mu , \\ V \left( t_{n+1} \right)= & {} V \left( t_0 \right) + \frac{\tau t^{\tau - 1}_n \left( 1 - \eta \right) }{ AB\left( \eta \right) } F_7 \left( t_n, V \left( t_n \right) \right) + \frac{\eta \tau }{AB \left( \eta \right) \Gamma \left( \eta \right) } \sum _{k=0}^{n} \int _{t_k}^{t_{k+1}} \mu ^{\tau - 1} \left( t_{n+1} - \mu \right) ^{\eta - 1} F_7 \left( \mu , V \left( \mu \right) \right) d \mu , \\ R \left( t_{n+1} \right)= & {} R \left( t_0 \right) + \frac{\tau t^{\tau - 1}_n \left( 1 - \eta \right) }{ AB\left( \eta \right) } F_8 \left( t_n, R \left( t_n \right) \right) + \frac{\eta \tau }{AB \left( \eta \right) \Gamma \left( \eta \right) } \sum _{k=0}^{n} \int _{t_k}^{t_{k+1}} \mu ^{\tau - 1} \left( t_{n+1} - \mu \right) ^{\eta - 1} F_8 \left( \mu , R \left( \mu \right) \right) d \mu , \end{aligned}$$

**Figure 7:**
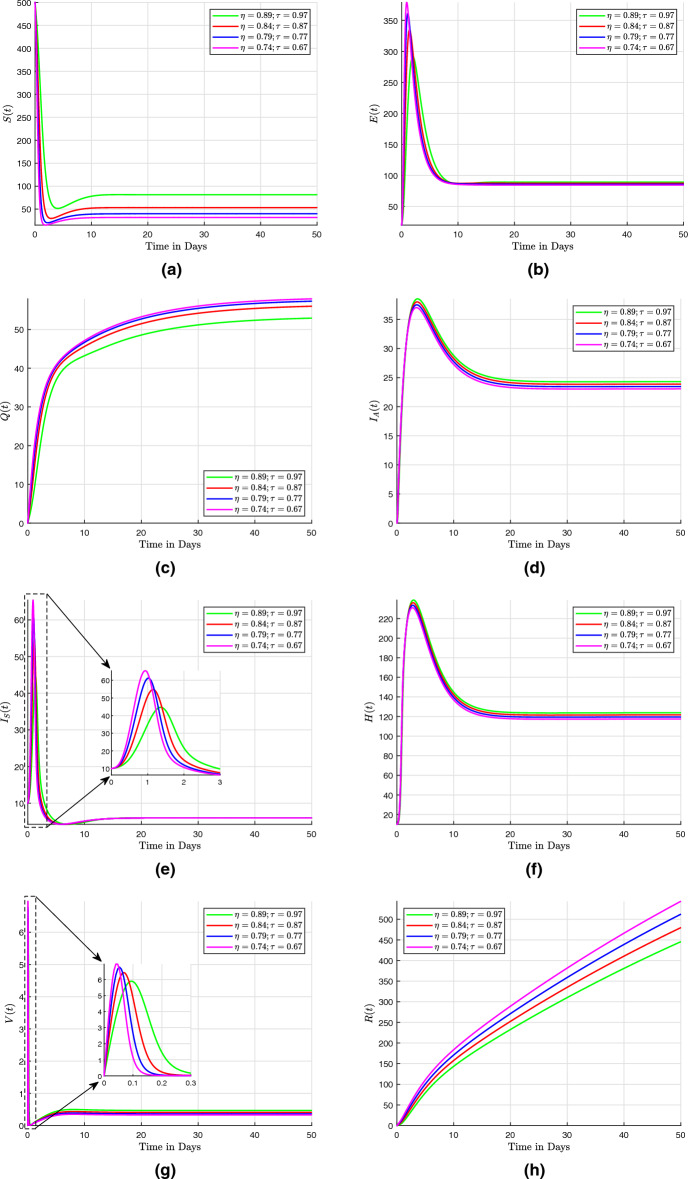
The dynamical behavior of the (**a**) susceptible population density; (**b**) exposed population density; (**c**) quarantined population density; (**d**) asymptomatically infected population density; (**e**) symptomatically infected population density; (**f**) hospitalized population density; (**g**) vaccinated population density; and (**h**) recovered population density; for fractal-fractional derivative in the Atangana–Baleanu sense.

Approximating $\mu ^{\tau - 1} F \left( \mu , W \left( \mu \right) \right)$ for $i = 1, 2, 3, 4, 5, 6, 7, 8$ and $W = \left[ S, E, Q, I_A, I_S, H, V, R \right] ^T$ in $\left[ t_k , t_{k+1} \right]$ as presented earlier, the following numerical scheme was developed by us.

$$\begin{aligned} S \left( t_{n+1} \right)= & {} S \left( t_0 \right) + \frac{\tau t^{\tau - 1}_n \left( 1 - \eta \right) }{ AB\left( \eta \right) } F_1 \left( t_n, S \left( t_n \right) \right) + \frac{\tau \left( \Delta t \right) ^\eta }{AB \left( \eta \right) \Gamma \left( \eta + 2 \right) } \sum _{k=0}^{n} \left[ t^{\tau - 1}_k F_1 \left( t_k, S \left( t_k \right) \right) \left( \left( n + 1 - k \right) ^\eta \left( n - k + 2 \eta \right) \right. \right. \\ {}{} & {} \left. \left. - \left( n - k \right) ^\eta \left( n - k + 2 + 2 \eta \right) \right) - t^{\tau - 1}_{k-1} F_1 \left( t_{k-1}, S \left( t_{k-1} \right) \right) \left( \left( n + 1 - k \right) ^{\eta + 1} - \left( n - k \right) ^\eta \left( n - k + 1 + \eta \right) \right) \right] , \\ E \left( t_{n+1} \right)= & {} E \left( t_0 \right) + \frac{\tau t^{\tau - 1}_n \left( 1 - \eta \right) }{ AB\left( \eta \right) } F_2 \left( t_n, E \left( t_n \right) \right) + \frac{\tau \left( \Delta t \right) ^\eta }{AB \left( \eta \right) \Gamma \left( \eta + 2 \right) } \sum _{k=0}^{n} \left[ t^{\tau - 1}_k F_2 \left( t_k, E \left( t_k \right) \right) \left( \left( n + 1 - k \right) ^\eta \left( n - k + 2 \eta \right) \right. \right. \\ {}{} & {} \left. \left. - \left( n - k \right) ^\eta \left( n - k + 2 + 2 \eta \right) \right) - t^{\tau - 1}_{k-1} F_2 \left( t_{k-1}, E \left( t_{k-1} \right) \right) \left( \left( n + 1 - k \right) ^{\eta + 1} - \left( n - k \right) ^\eta \left( n - k + 1 + \eta \right) \right) \right] , \\ Q \left( t_{n+1} \right)= & {} Q \left( t_0 \right) + \frac{\tau t^{\tau - 1}_n \left( 1 - \eta \right) }{ AB\left( \eta \right) } F_3 \left( t_n, Q \left( t_n \right) \right) + \frac{\tau \left( \Delta t \right) ^\eta }{AB \left( \eta \right) \Gamma \left( \eta + 2 \right) } \sum _{k=0}^{n} \left[ t^{\tau - 1}_k F_3 \left( t_k, Q \left( t_k \right) \right) \left( \left( n + 1 - k \right) ^\eta \left( n - k + 2 \eta \right) \right. \right. \\ {}{} & {} \left. \left. - \left( n - k \right) ^\eta \left( n - k + 2 + 2 \eta \right) \right) - t^{\tau - 1}_{k-1} F_3 \left( t_{k-1}, Q \left( t_{k-1} \right) \right) \left( \left( n + 1 - k \right) ^{\eta + 1} - \left( n - k \right) ^\eta \left( n - k + 1 + \eta \right) \right) \right] , \\ I_A \left( t_{n+1} \right)= & {} I_A \left( t_0 \right) + \frac{\tau t^{\tau - 1}_n \left( 1 - \eta \right) }{ AB\left( \eta \right) } F_4 \left( t_n, I_A \left( t_n \right) \right) + \frac{\tau \left( \Delta t \right) ^\eta }{AB \left( \eta \right) \Gamma \left( \eta + 2 \right) } \sum _{k=0}^{n} \left[ t^{\tau - 1}_k F_4 \left( t_k, I_A \left( t_k \right) \right) \left( \left( n + 1 - k \right) ^\eta \left( n - k + 2 \eta \right) \right. \right. \\ {}{} & {} \left. \left. - \left( n - k \right) ^\eta \left( n - k + 2 + 2 \eta \right) \right) - t^{\tau - 1}_{k-1} F_4 \left( t_{k-1}, I_A \left( t_{k-1} \right) \right) \left( \left( n + 1 - k \right) ^{\eta + 1} - \left( n - k \right) ^\eta \left( n - k + 1 + \eta \right) \right) \right] , \\ I_S \left( t_{n+1} \right)= & {} I_S \left( t_0 \right) + \frac{\tau t^{\tau - 1}_n \left( 1 - \eta \right) }{ AB\left( \eta \right) } F_5 \left( t_n, I_S \left( t_n \right) \right) + \frac{\tau \left( \Delta t \right) ^\eta }{AB \left( \eta \right) \Gamma \left( \eta + 2 \right) } \sum _{k=0}^{n} \left[ t^{\tau - 1}_k F_5 \left( t_k, I_S \left( t_k \right) \right) \left( \left( n + 1 - k \right) ^\eta \left( n - k + 2 \eta \right) \right. \right. \\ {}{} & {} \left. \left. - \left( n - k \right) ^\eta \left( n - k + 2 + 2 \eta \right) \right) - t^{\tau - 1}_{k-1} F_5 \left( t_{k-1}, I_S \left( t_{k-1} \right) \right) \left( \left( n + 1 - k \right) ^{\eta + 1} - \left( n - k \right) ^\eta \left( n - k + 1 + \eta \right) \right) \right] , \\ H \left( t_{n+1} \right)= & {} H \left( t_0 \right) + \frac{\tau t^{\tau - 1}_n \left( 1 - \eta \right) }{ AB\left( \eta \right) } F_6 \left( t_n, H \left( t_n \right) \right) + \frac{\tau \left( \Delta t \right) ^\eta }{AB \left( \eta \right) \Gamma \left( \eta + 2 \right) } \sum _{k=0}^{n} \left[ t^{\tau - 1}_k F_6 \left( t_k, H \left( t_k \right) \right) \left( \left( n + 1 - k \right) ^\eta \left( n - k + 2 \eta \right) \right. \right. \\ {}{} & {} \left. \left. - \left( n - k \right) ^\eta \left( n - k + 2 + 2 \eta \right) \right) - t^{\tau - 1}_{k-1} F_6 \left( t_{k-1}, H \left( t_{k-1} \right) \right) \left( \left( n + 1 - k \right) ^{\eta + 1} - \left( n - k \right) ^\eta \left( n - k + 1 + \eta \right) \right) \right] , \\ V \left( t_{n+1} \right)= & {} V \left( t_0 \right) + \frac{\tau t^{\tau - 1}_n \left( 1 - \eta \right) }{ AB\left( \eta \right) } F_7 \left( t_n, V \left( t_n \right) \right) + \frac{\tau \left( \Delta t \right) ^\eta }{AB \left( \eta \right) \Gamma \left( \eta + 2 \right) } \sum _{k=0}^{n} \left[ t^{\tau - 1}_k F_7 \left( t_k, V \left( t_k \right) \right) \left( \left( n + 1 - k \right) ^\eta \left( n - k + 2 \eta \right) \right. \right. \\ {}{} & {} \left. \left. - \left( n - k \right) ^\eta \left( n - k + 2 + 2 \eta \right) \right) - t^{\tau - 1}_{k-1} F_7 \left( t_{k-1}, V \left( t_{k-1} \right) \right) \left( \left( n + 1 - k \right) ^{\eta + 1} - \left( n - k \right) ^\eta \left( n - k + 1 + \eta \right) \right) \right] , \\ R \left( t_{n+1} \right)= & {} R \left( t_0 \right) + \frac{\tau t^{\tau - 1}_n \left( 1 - \eta \right) }{ AB\left( \eta \right) } F_8 \left( t_n, R \left( t_n \right) \right) + \frac{\tau \left( \Delta t \right) ^\eta }{AB \left( \eta \right) \Gamma \left( \eta + 2 \right) } \sum _{k=0}^{n} \left[ t^{\tau - 1}_k F_8 \left( t_k, R \left( t_k \right) \right) \left( \left( n + 1 - k \right) ^\eta \left( n - k + 2 \eta \right) \right. \right. \\ {}{} & {} \left. \left. - \left( n - k \right) ^\eta \left( n - k + 2 + 2 \eta \right) \right) - t^{\tau - 1}_{k-1} F_8 \left( t_{k-1}, R \left( t_{k-1} \right) \right) \left( \left( n + 1 - k \right) ^{\eta + 1} - \left( n - k \right) ^\eta \left( n - k + 1 + \eta \right) \right) \right] , \end{aligned}$$

The Atangana–Beleanu model allows for a more accurate representation of complex systems with non-integer dimensions, providing insights into the dynamics of population growth and decline. By incorporating fractal-fractional derivatives, the model can capture the intricate patterns and interactions within populations that traditional calculus may overlook. This method gets closer to a more accurate picture of how populations change over time as illustrated in Fig. 7, which lets us make more accurate predictions and do more in-depth research. Furthermore, it offers a fresh viewpoint on how ecological modelling incorporates fractal geometry and fractional calculus. This approach simplifies the demonstration of the intricate dynamics of disease transmission, which can aid in the development of novel therapeutic and preventative strategies. By incorporating fractal geometry and fractional calculus into ecological modeling, researchers can better understand the complex dynamics of population changes. This approach not only enhances our ability to predict population trends more accurately but also provides valuable insights for improving public health interventions.

## Comparison

**Figure 8:**
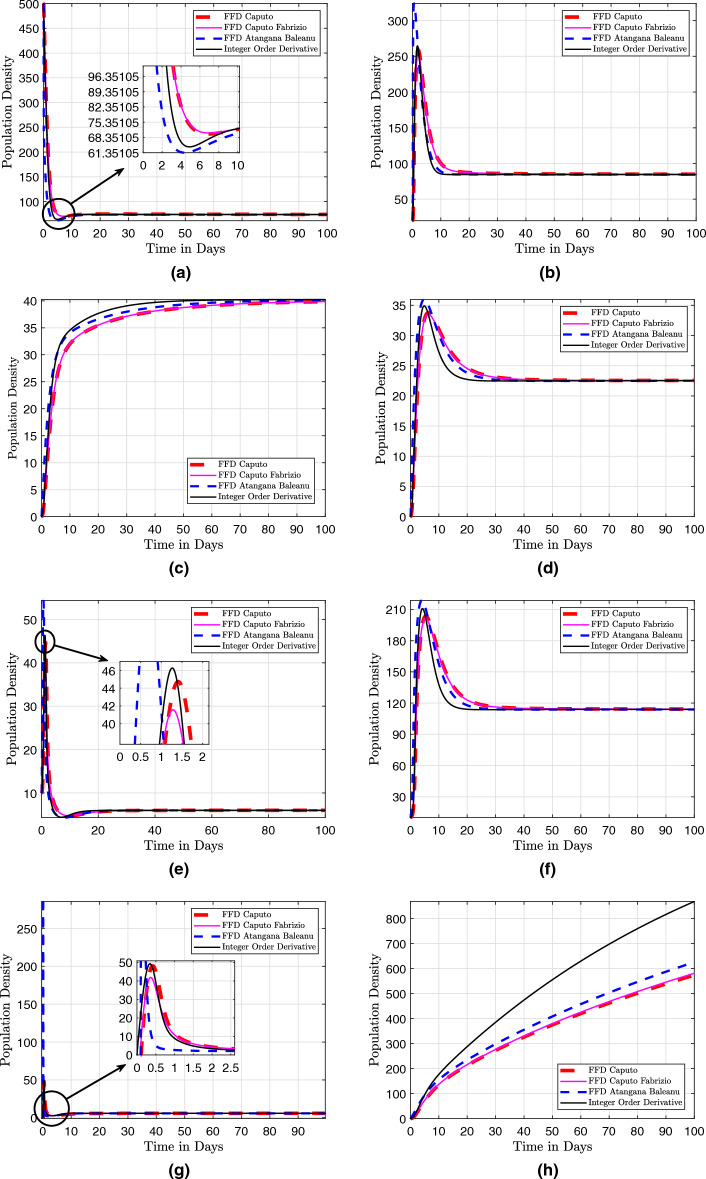
Comparison of the (**a**) susceptible population density; (**b**) exposed population density; (**c**) quarantined population density; (**d**) asymptomatically infected population density; (**e**) symptomatically infected population density; (**f**) hospitalized population density; (**g**) vaccinated population density; and (**h**) recovered population density; for $\eta = 0.5, \tau = 0.47$ using fractal-fractional differential operator in the Caputo, Caputo Fabrizio, and Atangana–Baleanu sense.

The deterministic mathematical model of SARS-COV-2 with fractal-fractional operators is compared in Fig. 8 to other models that have already been used to study the virus. The results show that the fractal-fractional model provides a more accurate representation of the virus’s behavior, allowing for better predictions and understanding. This comparison highlights the importance of incorporating fractal-fractional operators in mathematical models for infectious diseases like SARS-COV-2. The purpose of this comparative study is to assess the efficacy and precision of the suggested model in forecasting the trajectory and consequences of SARS-COV-2 in Pakistan. By analyzing the data and outcomes, researchers can better prepare for potential future outbreaks and implement more effective control measures. This study contributes to the ongoing efforts to improve public health responses to infectious diseases by utilizing advanced mathematical modeling techniques. Understanding the special qualities and benefits of this particular model-especially in the context of Pakistan-is the main goal of the study. Using fractal and fractional operators-which have been demonstrated to be effective at capturing complex dynamics-the deterministic mathematical model of SARS-COV-2 with fractal-fractional operators adopts a novel strategy. This innovative approach allows for a more accurate representation of the virus’s spread and potential impact on different populations. By incorporating these advanced techniques, public health officials can make more informed decisions when developing strategies to prevent and control future outbreaks. In order to better understand this model’s capacity to make accurate predictions tailored to the Pakistani context, this comparative study will compare it to other well-established models and examine its advantages and disadvantages. It also looks for ways to compare the fractal-fractional operator approach to other models and determine any benefits or drawbacks that might exist. These kinds of insights are important for planning future studies and policy initiatives. By evaluating the effectiveness of different models, researchers can identify the most reliable tools for predicting and managing outbreaks in Pakistan. This research can ultimately contribute to more effective public health interventions and strategies in the region.

An important strategy in the investigation of fractal-fractional models for comprehending the dynamics of COVID-19 is the application of fractal-fractional calculus. To analyze the intricate mechanisms and dissemination of SAR-CoV-2, these derivatives are employed. This study has shown promising outcomes in capturing the complex virus behavior. It is worth mentioning that other fractal-fractional derivatives, like Caputo derivatives in general, have also produced satisfactory results, even though Caputo–Fabrizio is efficient. The efficacy of Caputo–Fabrizio derivatives is demonstrated in this study by introducing a fractal-fractional order model for COVID-19 transmission. A significant variation can be found in the rate of convergence. Rapid convergence is a characteristic of Caputo–Fabrizio models, which increases their applicability for rapid analysis. Contrarily, the convergence process for alternative fractal-fractional derivatives might be prolonged. The particular demands of the modeling undertaking should inform the evaluation of this variation in convergence rates. Though other fractal-fractional derivatives also perform admirably, Caputo–Fabrizio is particularly noteworthy for its rapid convergence and efficacy. The criteria and requirements of the modeling scenario dictate which one is used. The choice ultimately depends on the specific needs and objectives of the modeling scenario. The future of modeling scenarios will likely continue to evolve and adapt as technology and research progress. We hope this comparetive analysis will provide valuable insights and inform the decision-making process for future modeling scenarios.

## Results and discussion

**Figure 9:**
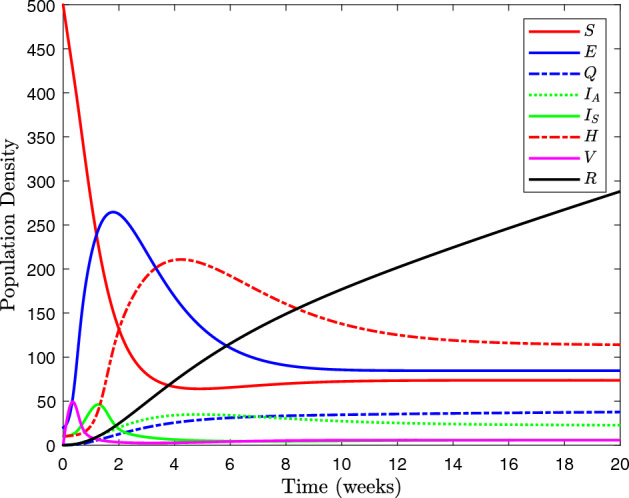
Dynamic behaviour of population densities, taking into account the influence of hospitals and the vaccination campaign.

Here, we examine how well the model (10) has been performing quantitatively in relation to the current vaccination drive. The major objective of this research is to determine what helps people who have contracted the COVID-19 virus recover. This will pave the way for future preventative measures, which will lessen the virus’s negative impact and lessen the domino effect of its continuous mutation and spread. Interpreting the numerical results’ implications for the model is the aim of this analysis. This entails examining the effects of various preventive measures, such as vaccination, isolation, and potential hospital admission or patient relocation strategies for those exhibiting more severe symptoms. In addition to receiving symptom-specific care, patients can receive a booster vaccination by receiving the proper medical attention in an appropriate setting. It is assumed that the baseline population densities for different compartments are as follows: $S=500, E=20, Q=0, I_{A}=0, I_{S}=10, H=10, V=0,$ and $R=0$. Figure 9 illustrates the population dynamics and densities in the model. The graph indicates that susceptibility is progressively decreasing as time passes and various preventative measures are considered. Especially if the virus spreads more quickly and the number of people exposed to it keeps growing. The amount of coronavirus viruses in the environment will keep increasing over the coming days, increasing the likelihood that people may become infected. This means that increasing the hospitalization rate is urgently and critically necessary. Which may be the cause and result in a reduction in the symptoms caused by the virus in the surroundings. As a result, there is a decline in the number of people who are symptomatically infected in society (Supplementary Information).

The findings of this study suggest that administering a booster vaccine dose during hospitalization leads to the greatest degree of disease resistance and a notable implementation of infection control measures. This intervention promotes accelerated healing in individuals and reduces a minimal increase in mortality rates associated with the condition. The results of our study depend on both the signs of the virus and the degree of acceptability of the vaccination within the community. It is especially important to ensure disease protection for those who are having severe symptoms since they require more protection. The need of putting public safety measures first, though, cannot be overstated. The need for mass vaccination is essential for achieving significant levels of population immunity. Furthermore, those who are hospitalized as well as those who display the highest susceptibility require the administration of a booster dose.

It is imperative, from a public health standpoint, to possess knowledge regarding the determinants of hospitalization rates and vaccine hesitancy in order to identify subgroups with lower-than-anticipated vaccination rates and prolonged recovery periods following infection. It is suggested that the inclusion of symptomatically infected persons in hospital settings will play a crucial role in the sustained management and eradication of the COVID-19 within the surrounding environment. Conversely, historical precedent in dealing with various diseases has demonstrated the arduous nature of disease eradication, typically necessitating a comprehensive and multifaceted approach. This assertion has been substantiated by prior empirical evidence. It is expected that a similar scenario would unfold with respect to COVID-19, as the elimination of the virus is expected to provide significant difficulties in the foreseeable future, necessitating the formulation of a global approach. Although the implementation of widespread vaccination, quarantine measures for susceptible individuals, and hospitalization of symptomatic cases is expected to significantly decrease the prevalence of social transmission and virus-related symptoms in society, the introduction of a booster dose, which reduces the recovery time, may still present a possibility for the virus to cease spreading.

**Figure 10:**
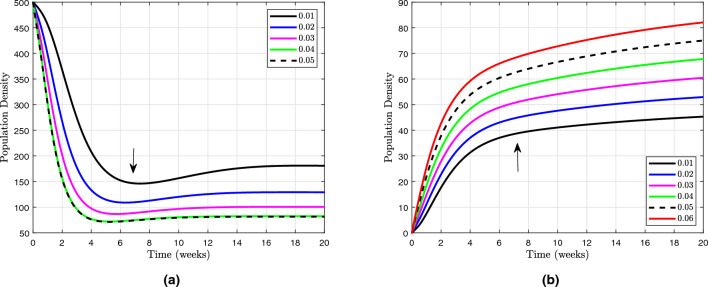
Population densities of (**a**) susceptible peoples and (**b**) quarantined peoples, for various values of $\beta$.

**Figure 11:**
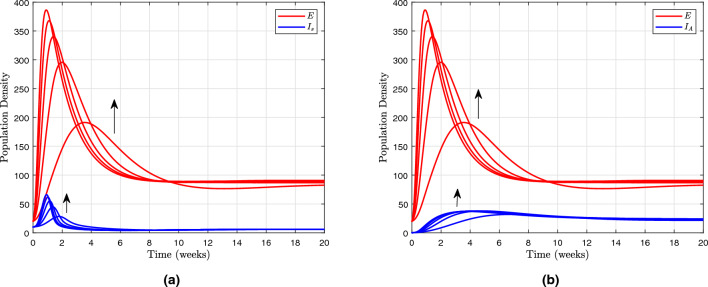
Population densities of exposed peoples vs. (**a**) symptomatically infected people for different values of $\omega$ and (**b**) asymptomatically infected peoples for various values of *f*.

The effect of the parameter $\beta$ on sensitive and quarantined people is shown in Fig. 10, which shows that when the rate of quarantined people goes up, the density of sensitive people drops quickly. This suggests that increasing the parameter $\beta$ leads to a more efficient containment of the virus, as more individuals are being quarantined. Additionally, the decrease in the density of sensitive people indicates a potential decrease in the overall spread of the virus within the population. Those who have been exposed to the virus as well as those who have contracted the disease can see the effect of the parameter $\omega$ in Fig. 11a. This illustrates that as $\omega$ increases from 0.002 to 0.01, the $I_{S}$ and *E* populations increase as well, with a lesser rate of symptomatically infected people as compared to exposed population density. This suggests that a higher value of $\omega$ leads to a higher proportion of individuals being exposed to the virus without developing symptoms. This information is crucial for understanding the potential impact of different values of $\omega$ on the transmission dynamics and control measures of the virus. Figure 11b shows how the parameter *f* affects the proportion of infected and exposed individuals. The population of *E* rises as the parameter *f* is increased from 0.01 to 0.018, whereas the population density of $I_{A}$ falls and might even be abolished with perfect protective plaining. These findings suggest that increasing the parameter *f* can lead to a higher number of individuals becoming exposed to the virus, potentially resulting in a larger outbreak. However, it is important to note that with perfect protective planning, the population density of symptomatic infected individuals ($I_{A}$) can be significantly reduced or even eliminated entirely.

**Figure 12:**
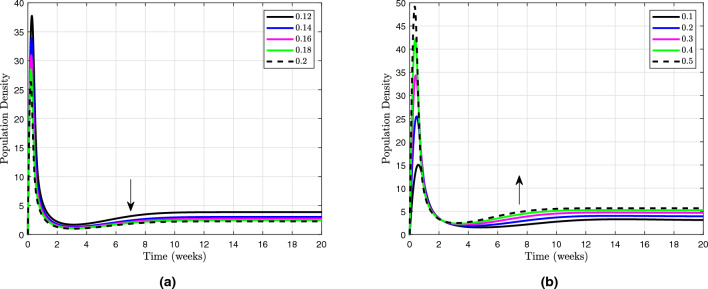
Population densities of vaccinated peoples (**a**) for different values of *m* and (**b**) for various values of *p*.

Taking the values of *p* and *m* into account, we looked at the changes in the density of the vaccinated population in other Fig. 12. According to these figures, the number of people who have been vaccinated drops right away when the parameter *p* is changed from 0.1 to 0.2, while the number of people who have been vaccinated starts to rise when the parameter *m* is changed from 0.1 to 0.5 in the first week. This suggests that the parameter *p* has a significant impact on the initial uptake of vaccination, with a higher value leading to a decrease in the number of vaccinated individuals. On the other hand, the parameter *m* seems to play a role in increasing vaccination rates over time, as evidenced by the rise in vaccinated population when its value is increased.

**Figure 13:**
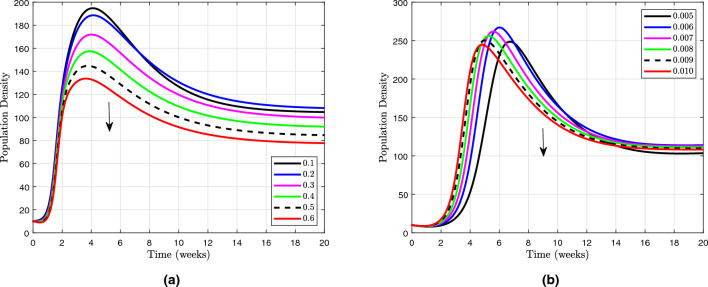
Population densities of hospitalized peoples (**a**) for different values of $h_1$ and (**b**) for various values of $h_2$.

**Figure 14:**
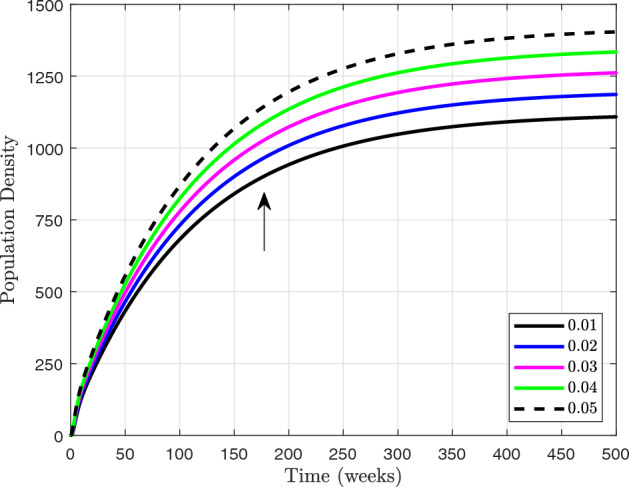
Population densities of recovered individuals for various values of $\alpha$.

The impact of the parameter $h_1$ on symptomatically infected individuals is depicted in Fig. 13a. Which influences that sending/admitted in hospital to symptomatically infected individuals is necessary for full recovery from the COVID-19 together with effective control of the virus. This finding suggests that providing adequate medical care and resources to symptomatically infected individuals can significantly contribute to their recovery. Additionally, it highlights the importance of implementing measures to control the spread of the virus in order to prevent further transmission and protect vulnerable populations. The effect that $h_2$ has on hospitalised individuals is depicted in Fig. 13b. This influence is necessary for full recovery from the COVID-19 together with effective control of the virus. Which demonstrates that the standard procedure for the booster dose for symptomatic patients who are hospitalized should involve a rise in the booster shots during the first few days, followed by a gradual decrease thereafter. This approach ensures that the hospitalized individuals receive a higher concentration of the booster dose initially, which helps in combating the virus more effectively. As their condition improves, a gradual decrease in booster shots can be implemented to maintain the necessary level of protection without overwhelming their immune system. This tailored approach aims to optimize the recovery process and minimize the risk of transmission within healthcare settings. Figure 14 shows how the density of the recovered population is affected by the parameter $\alpha$. Along with the increase in the value of the parameter from 0.01 to 0.05, there is also an increase in the number of recoveries that have occurred. This suggests that a higher value of the parameter $\alpha$ leads to a more effective recovery process. However, it is important to strike a balance as an excessively high value may also result in an overwhelming number of recoveries, straining healthcare resources.

## Conclusion

To summarize, our investigation utilizes an innovative fractal-fractional mathematical model to demonstrate the complex dynamics of the SARS-CoV-2 virus in Pakistan. The exploration of the interplay between COVID-19 and vaccination using FDEs provides valuable insights. By considering various scenarios and parameters, we can gain a better understanding of the impact of vaccination on the spread of the virus. This information can inform policymakers and healthcare professionals in their decision-making processes regarding vaccination strategies and control measures. The paper incorporates a comprehensive analysis of the proposed mathematical model’s behavior, including stability considerations, basic reproduction numbers, and equilibrium points. Additionally, it discusses the impact of different vaccination strategies and their effectiveness in controlling the spread of the virus. It also examines the sensitivity of the model to various parameters and provides recommendations for future research.

The study introduces a comparative analysis of the general fractional model’s fit to real data. The findings reveal a specific case of the general fractional formula providing a superior fit compared to classical models. This emphasizes the importance of considering fractional approaches for a more accurate representation of dynamic systems. Additionally, it contributes to the understanding of COVID-19 spread by formulating a deterministic fractal-fractional order model. The establishment of the existence and uniqueness of solutions adds depth to the modeling of the pandemic’s dynamics. This deterministic approach provides a valuable perspective for capturing the uncertainties inherent in infectious disease dynamics. The deterministic analysis enhances our comprehension of the complex dynamics involved. This contributes to the growing body of research aiming to decipher the intricate patterns of COVID-19 transmission.

Thus, our research makes a valuable contribution to the collective endeavors of the scientific community in their struggle against the COVID-19 pandemic. Using mathematical modeling, fractal-fractional operators, and real-world data analysis together helps us understand how the virus changes over time and come up with evidence-based ways to keep it from hurting people’s health. It is hypothesized that the scientific advantages described above will facilitate the progression of pandemic modeling and response strategies. This will ultimately aid in mitigating the impact of future outbreaks and saving lives.

The success of the general fractional model in fitting real data suggests the need for further exploration of its applications in various fields. Future research could investigate its efficacy in modeling other dynamic systems and phenomena beyond infectious diseases. Building on the comprehensive review of epidemic models using fractional calculus, future directions may involve further integration and exploration of fractional calculus in studying emerging diseases. This approach could contribute to a more unified understanding of complex disease dynamics.

### Supplementary Information

Supplementary Information.

## Data Availability

All data used in this study can be accessed directly through the dashboard of the Public Health Advisory Platform by Ministry of National Health Services Regulations and Coordinations^20^, with all parameter values provided in the text of the manuscript.

## Abbreviations

COVID-19: Corona virus disease 2019
FDEs: Fractional ordinary differential equations
PEG: Poly(ethylene glycol)
MERS-CoV: Middle east respiratory syndrome corona-virus
SARS-CoV: Severe acute respiratory syndrome corona-virus
EVBC: European Virus Bioinformatics Center
PRCC: Partial rank correlation coefficient
FFD: Fractal fractional derivative

## References

[1] World Health Organization. https://www.who.int/emergencies/diseases/novel-coronavirus-2019/question-and-answers-hub/q-a-detail/coronavirus-disease-covid-19. https://www.who.int/novel-corona-virus-2019. Accessed 11 May 2020.

[2] Bennett, G., Young, E., Butler, I. & Coe, S. The impact of lockdown during the COVID-19 outbreak on dietary habits in various population groups: A scoping review. *Front. Nutr.* **8**, 626432 (2021). 33748175 10.3389/fnut.2021.626432 PMC7969646

[3] Di Domenico, L., Pullano, G., Sabbatini, C. E., Boëlle, P. Y. & Colizza, V. Impact of lockdown on COVID-19 epidemic in Île-de-France and possible exit strategies. *BMC Med.* **18**(1), 1–13 (2020). 32727547 10.1186/s12916-020-01698-4 PMC7391016

[4] Le, T. T. *et al.* The COVID-19 vaccine development landscape. *Nat. Rev. Drug Discov.* **19**(5), 305–306 (2020). 32273591 10.1038/d41573-020-00073-5

[5] Grzybowski, J. M. V., Da Silva, R. V. & Rafikov, M. Expanded SEIRCQ model applied to COVID-19 epidemic control strategy design and medical infrastructure planning. *Math. Probl. Eng.* **20**, 20 (2020).

[6] Jahanshahi, H., Sajjadi, S. S., Bekiros, S. & Aly, A. A. On the development of variable-order fractional hyperchaotic economic system with a nonlinear model predictive controller. *Chaos Solitons Fractals* **144**, 110698 (2021). 10.1016/j.chaos.2021.110698

[7] Center for Disease Control and Prevention (CDC). https://covid.cdc.gov/covid-data-tracker/#datatracker-home. https://www.cdc.gov/corona-virus. Accessed 3 Aug 2021.

[8] Jin, F., Qian, Z. S., Chu, Y. M. & Ur Rahman, M. On nonlinear evolution model for drinking behavior under Caputo-Fabrizio derivative. *J. Appl. Anal. Comput.* **12**(2), 790–806 (2022).

[9] Kubra, K. T., Gulshan, S. & Ali, R. An Atangana–Baleanu derivative-based fractal-fractional order model for the monkey pox virus: A case study of USA. *Partial Differ. Equ. Appl. Math.* **20**, 100623 (2024). 10.1016/j.padiff.2024.100623

[10] Shah, K. *et al.* Fractal-fractional mathematical model addressing the situation of corona virus in Pakistan. *Results Phys.* **19**, 103560 (2020). 33200064 10.1016/j.rinp.2020.103560 PMC7658553

[11] Li, B., Zhang, T. & Zhang, C. Investigation of financial bubble mathematical model under fractal-fractional Caputo derivative. *FRACTALS (fractals)* **31**(05), 1–13 (2023).

[12] Li, P. *et al.* Dynamical properties of a meminductor chaotic system with fractal-fractional power law operator. *Chaos Solitons Fractals* **175**, 114040 (2023). 10.1016/j.chaos.2023.114040

[13] Fatima, B., Yavuz, M., Ur Rahman, M. & Al-Duais, F. S. Modeling the epidemic trend of middle eastern respiratory syndrome coronavirus with optimal control. *Math. Biosci. Eng.* **20**(7), 11847–11874 (2023). 37501423 10.3934/mbe.2023527

[14] Fatima, B., Yavuz, M., Ur Rahman, M., Althobaiti, A. & Althobaiti, S. Predictive modeling and control strategies for the transmission of middle east respiratory syndrome coronavirus. *Math. Comput. Appl.* **28**(5), 98 (2023).

[15] Kubra, K. T. & Ali, R. Modeling and analysis of novel COVID-19 outbreak under fractal-fractional derivative in Caputo sense with power-law: A case study of Pakistan. *Model. Earth Syst. Environ.* **20**, 1–18 (2023). 10.1007/s40808-023-01747-w PMC10019432 37361699

[16] Atangana, A. & Qureshi, S. Modeling attractors of chaotic dynamical systems with fractal-fractional operators. *Chaos Solitons Fractals* **123**, 320–337 (2019). 10.1016/j.chaos.2019.04.020

[17] Ahmed, I., Modu, G. U., Yusuf, A., Kumam, P. & Yusuf, I. A mathematical model of corona-virus Disease (COVID-19) containing asymptomatic and symptomatic classes. *Results Phys.* **21**, 103776 (2021). 33432294 10.1016/j.rinp.2020.103776 PMC7787076

[18] Tartof, S. Y. *et al.* Effectiveness of a third dose of BNT162b2 mRNA COVID-19 vaccine in a large US health system: A retrospective cohort study. *Lancet Region. Health-Am.* **9**, 100198 (2022). 10.1016/j.lana.2022.100198 PMC8841530 35187521

[19] Hethcote, H. W. The mathematics of infectious diseases. *SIAM Rev.* **42**(4), 599–653 (2000). 10.1137/S0036144500371907

[20] Pakistan recorded corona-virus Cases since the epidemic began, according to the COVID-19 health advisory platform by ministry of national health services regulations and coordinations. Government of Pakistan. Accessed 21 May 2020.

[21] Diekmann, O., Heesterbeek, J. A. P. & Metz, J. A. On the definition and the computation of the basic reproduction ratio in models for infectious diseases in heterogeneous populations. *J. Math. Biol.* **28**(4), 365–382 (1990). 2117040 10.1007/BF00178324

[22] Obasi, C. & Mbah, G. C. E. On the stability analysis of a mathematical model of lassa fever disease dynamics. *J. Nigerian Soc. Math. Biol.* **2**, 135–144 (2019).

[23] Van den Driessche, P. & Watmough, J. Reproduction numbers and sub-threshold endemic equilibria for compartmental models of disease transmission. *Math. Biosci.* **180**(1–2), 29–48 (2002). 12387915 10.1016/S0025-5564(02)00108-6

[24] Caputo, M. Linear models of dissipation whose Q is almost frequency independent-II. *Geophys. J. Int.* **13**(5), 529–539 (1967). 10.1111/j.1365-246X.1967.tb02303.x

[25] Caputo, M. & Fabrizio, M. A new definition of fractional derivative without singular kernel. *Progress Fract. Differ. Appl.* **1**(2), 73–85 (2015).

[26] Atangana, A., & Baleanu, D. New fractional derivatives with nonlocal and non-singular kernel: Theory and application to heat transfer model. arXiv:1602.03408 (arXiv preprint) (2016).

[27] Arif, M., Kumam, P., Kumam, W., Akgul, A. & Sutthibutpong, T. Analysis of newly developed fractal-fractional derivative with power law kernel for MHD couple stress fluid in channel embedded in a porous medium. *Sci. Rep.* **11**(1), 20858 (2021). 34675245 10.1038/s41598-021-00163-3 PMC8531019

[28] Ali, Z., Rabiei, F., Shah, K. & Khodadadi, T. Qualitative analysis of fractal-fractional order COVID-19 mathematical model with case study of Wuhan. *Alex. Eng. J.* **60**(1), 477–489 (2021). 10.1016/j.aej.2020.09.020

[29] Arfan, M., Shah, K. & Ullah, A. Fractal-fractional mathematical model of four species comprising of prey-predation. *Phys. Scr.* **96**(12), 124053 (2021). 10.1088/1402-4896/ac2f37

[30] Ghanbari, B. & Gómez-Aguilar, J. F. Analysis of two avian influenza epidemic models involving fractal-fractional derivatives with power and Mittag–Leffler memories. *Chaos Interdiscip. J. Nonlinear Sci.* **29**(12), 123113 (2019). 10.1063/1.5117285 31893661

[31] Jeelani, M. B. *et al.* Mathematical modeling and forecasting of COVID-19 in Saudi Arabia under fractal-fractional derivative in caputo sense with power-law. *Axioms* **10**(3), 228 (2021). 10.3390/axioms10030228

[32] Qu, H. *et al.* Investigating fractal-fractional mathematical model of tuberculosis (TB) under fractal-fractional caputo operator. *Fractals* **30**(05), 2240126 (2022). 10.1142/S0218348X22401260

[33] Sweilam, N. H., Al-Mekhlafi, S. M. & Almutairi, A. Fractal fractional optimal control for a novel malaria mathematical model; a numerical approach. *Results Phys.* **19**, 103446 (2020). 10.1016/j.rinp.2020.103446

