# Author Correction: Analysis and comparative study of a deterministic mathematical model of SARS-COV-2 with fractal-fractional operators: a case study

**DOI:** 10.1038/s41598-024-58414-y

**Published:** 2024-04-02

**Authors:** Khadija Tul Kubra, Rooh Ali, Rubayyi Turki Alqahtani, Samra Gulshan, Zahoor Iqbal

**Affiliations:** 1 Department of Mathematics, Government College University, Faisalabad, Punjab 38040 Pakistan; 2 Department of Mathematics and Statistics, College of Science, Imam Mohammad Ibn Saud Islamic University(IMSIU), Riyadh, Saudi Arabia; 3 School of Computer Science and Technology, Zhejiang Normal University, Jinhua, 321004 China

Correction to: *Scientific Reports* 10.1038/s41598-024-56557-6, published online 18 March 2024

The original version of this Article contained an error in Affiliation 3, which was incorrectly given as ‘College of Mathematics and Computer Science, Zhejiang Normal University, Jinhua, China’. The correct affiliation is listed below:

School of Computer Science and Technology, Zhejiang Normal University, Jinhua 321004, China.

The original Article has been corrected.

