# Supplementary material for: Analysis and comparative study of a deterministic mathematical model of SARS-COV-2 with fractal-fractional operators: a case study
Source: Sci Rep. 2024 Mar 18;14:6431. doi: 10.1038/s41598-024-56557-6 (PMC11335959; doi:10.1038/s41598-024-56557-6)
Supplement: Supplementary file 1 — Supplementary Information. [file 41598_2024_56557_MOESM1_ESM.pdf]

Appendix A

In mathematical modeling, accurately estimating unknown parameters is crucial for fitting models to observed data. MATLAB provides powerful tools for parameter estimation through optimization techniques. In this introduction, we'll walk through the fundamental steps of parameter estimation. By following below steps, readers can apply MATLAB's optimization tools to estimate unknown parameters and improve the fit of mathematical model to observed data.

1. Begin by defining the mathematical model that represents the relationship between the input variables and the observed data. In our case we use model 10 and 29. Here we explain each step of parameter estimations for model 10. Do all these step in the same manner for model 29 as well. Let us consider the model 10 with unknown parameters A and B (say).

2. Generate synthetic data using known or assumed values for the parameters. This data simulates the observations you might make in a real-world scenario, often including some level of noise to account for measurement errors. For this purpose, we first define the true parameters A and B. Then generate synthetic data using the model 10 with added random noise.
   Code:

```
t = [1, 2, 3, 4, 5];
true_A = 2;
true_B = 0.5;
observed_data = @model's_equations + 0.2 * randn(size(t));
```

3. Provide an initial guess for the unknown parameters. This guess serves as the starting point for the optimization algorithm to refine.
   Code:

```
initial_guess_A = 1;
initial_guess_B = 0.1;
initial_parameters = [initial_guess_A, initial_guess_B];
```

4. Create a cost function that quantifies the difference between the model predictions and the observed data. The goal is to minimize this cost function during the optimization process. For the model 10 just replace the the parameter A and B with "$params(1)$" and "$params(2)$" respectively in model 10.
   Code:

```
cost_function = @(params) Your_Model - observed_data;
```

   Here, "params" is a vector containing the parameters to be estimated (A and B).

5. Utilize an optimization algorithm, such as **lsqnonlin** in MATLAB, to iteratively adjust the parameters and minimize the cost function. This step involves finding the parameter values that result in the best fit to the observed data.
   Code:

```
options = optimset('Display', 'iter');
estimated_parameters = lsqnonlin(cost_function, initial_parameters, [], [], options);
```

   The **lsqnonlin** function minimizes the sum of squares of the elements of the vector returned by the cost function.

6. By using below code display both the true and estimated parameter values. This allows you to assess the accuracy of the estimation process.
   Code:

```
disp('True Parameters:');
disp(['A = ', num2str(true_A)]);
disp(['B = ', num2str(true_B)]);

disp('Estimated Parameters:');
disp(['A = ', num2str(estimated_parameters(1))]);
disp(['B = ', num2str(estimated_parameters(2))]);
```

7. Visualize the results by plotting the observed data alongside the fitted curve. This graphical representation helps in evaluating the goodness of fit.
   Code:

```
figure;
plot(t, observed_data, 'o', 'DisplayName', 'Observed Data');
hold on;
plot(t, Your_Model(estimated_parameters(.))), 'r-',...
'DisplayName', 'Fitted Curve');
```

8. Save the script and run it in MATLAB to execute all the steps.

9. If the results are not satisfactory, you may need to iterate through the process, adjusting the initial guess or refining other parameters.

By following these steps, you can apply MATLAB's optimization tools to estimate unknown parameters and improve the fit of your mathematical model to observed data. Readers can adapt this approach to more complex models and datasets by appropriately defining the cost function and choosing suitable optimization techniques.
